# Desiccation tolerance mechanisms in the green lineage and beyond

**DOI:** 10.1111/nph.71466

**Published:** 2026-07-24

**Authors:** Jenny Schuster, Robert VanBuren

**Affiliations:** ^1^ Plant Resilience Institute Michigan State University East Lansing MI 48824 USA; ^2^ Department of Plant Biology Michigan State University East Lansing MI 48824 USA; ^3^ Department of Plant, Soil, and Microbial Sciences Michigan State University East Lansing MI 48824 USA

**Keywords:** convergent evolution, desiccation tolerance, plant resilience, systems biology, terrestrialization

## Abstract

Water is essential for life on Earth, yet some plants can survive the near‐complete loss of cellular water, a trait known as desiccation tolerance. Although rare in vegetative tissues of vascular plants, desiccation tolerance is scattered across the plant kingdom, where it likely enabled early terrestrialization and supports survival in environments with prolonged or intermittent drying. Large‐scale multi‐omics, comparative, and functional studies over the past decade suggest that desiccation tolerance evolved primarily through the repurposing of ancient stress‐resilience mechanisms, giving rise to convergent phenotypes where cells are protected and stabilized during extreme dehydration. In photosynthetic lineages, desiccation is further compounded by light‐dependent photooxidative stress, requiring specialized mechanisms that safeguard the photosynthetic apparatus during both drying and recovery. Here, we synthesize our current understanding of the evolutionary origins and core mechanisms underlying plant desiccation tolerance and place them within a broader comparative context across kingdoms. We highlight how genome‐scale datasets suggest desiccation tolerance is an emergent, systems‐level phenotype coordinated across biological scales. Finally, we discuss the translational opportunities and constraints of desiccation tolerance in agriculture, medicine, biotechnology, and related fields.

## Graphical abstract

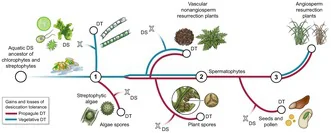

**Table:** 

	Contents	
	Summary	3159
I.	Introduction	3159
II.	Core mechanisms underlying desiccation tolerance	3161
III.	The emergent complexity of desiccation tolerance in plants	3166
IV.	Biotechnological applications of desiccation tolerance to crops and beyond	3169
V.	Concluding thoughts	3170
	Acknowledgements	3170
	References	3171

## I. Introduction

Life on Earth originated in water and nearly all biological macromolecules and cellular processes require a high internal water content to function (Womersley, 1981; Walters *et al*., 2005). Yet, water limitation is a pervasive challenge across many of Earth's biomes, and organisms have evolved a remarkable diversity of strategies to survive dehydration (Oliver *et al*., 2020). One of the most striking biological innovations for surviving water limitation is desiccation tolerance, or the ability to survive the near‐complete loss of cellular water (Bewley, 1979; Marks *et al*., 2025a). This trait has profoundly shaped the evolution of life on Earth. It enabled the recurrent colonization of land, particularly within the green lineage, collectively known as Viridiplantae, and supported the long‐term survival of seeds, pollen, spores, and other propagules (Hoekstra *et al*., 2001). Desiccation tolerance also underpins the stability of dried microbial cultures used in food production, biotechnology, and synthetic biology, and it facilitates the storage and distribution of pharmaceuticals (Potts *et al*., 2005; Marks *et al*., 2025a). Research on desiccation tolerance has grown rapidly in recent decades (Lotreck *et al*., 2024), yielding major advances in the genetic, physiological, biochemical, and systems‐level understanding of mechanisms underlying this remarkable trait. From an evolutionary perspective, desiccation tolerance has arisen repeatedly across deep timescales and throughout the tree of life, facilitating multiple independent transitions from aquatic to terrestrial environments and enabling survival during periodic drying across diverse habitats (Oliver *et al*., 2000; Alpert, 2005; Gaff & Oliver, 2013; Marks *et al*., 2021a). The ability to survive desiccation is widespread but sporadically distributed across archaea, bacteria, fungi, animal, and plant lineages (Oliver *et al*., 2000; Alpert, 2006; Leprince & Buitink, 2015).

Across nonplant lineages, desiccation tolerance is often restricted to resistant propagules, resting cells, or dormant stages specialized for persistence, whereas plant vegetative desiccation tolerance requires ordinary vegetative tissues to dry and then resume growth after rehydration. In animals, desiccation tolerance is found in certain invertebrates, such as tardigrades, nematodes, rotifers, brine shrimp cysts (*Artemia*), and the sleeping chironomid (*Polypedilum vanderplanki*; Crowe, 1971; Watanabe, 2006), all of which inhabit seasonal pools, soils, and surfaces that periodically dry. Many fungi produce desiccation‐tolerant propagules, including asexual conidia, sexual ascospores, and basidiospores, and sclerotia, which enable survival in fluctuating or seasonally dry environments (Kranner *et al*., 2008). In bacteria, extreme desiccation tolerance is most well‐studied in endospore‐forming Firmicutes, such as Bacillus and Clostridium, whose multilayered spore coats and DNA‐protective proteins allow persistence for decades or even centuries in a dormant state (Potts, 1994). Other bacterial taxa form cysts or other resistant cells that survive drying, including many cyanobacteria inhabiting desert crusts (Potts, 1999). Although desiccation tolerance is less studied in archaea, some halophilic and thermophilic species can persist through desiccation, often within salt crusts or biofilms, and may form cyst‐like resting cells under stress (Potts, 1994).

Within vascular plants, vegetative desiccation tolerance occurs in a small group of species known as ‘resurrection plants’, named for their seemingly miraculous recovery from near‐complete and typically lethal drying. Resurrection plants are often found in ecologically distinct but functionally convergent communities, such as rocky outcrops, marginal habitats in tropical and subtropical regions, and even quick‐drying surfaces of rocks or other plants in mesic environments (Gaff, 1977, 1987; Gaff & Bole, 1986; Porembski, 2007, 2011; Porembski *et al*., 2021; Smrithy *et al*., 2023). These environments experience rapid drying and rehydration cycles due to shallow soils, high irradiance, and erratic rainfall. Unlike ecosystems shaped by seasonal or prolonged drought, these habitats select for desiccation tolerance rather than drought avoidance strategies. They harbor diverse assemblages of desiccation‐tolerant organisms, including terrestrial algae, bryophytes, ferns, angiosperms, fungi, and microanimals that exhibit similar protective mechanisms despite vast evolutionary distances (Porembski & Barthlott, 2000; Porembski *et al*., 2021; Vanschoenwinkel *et al*., 2025). Although rare, resurrection plants are scattered across the plant phylogeny (Oliver *et al*., 2000; Wood, 2007; VanBuren *et al*., 2019; Marks *et al*., 2021a) and have become key models for studying both extreme stress adaptations and the evolution of complex traits.

**Fig. 1:**
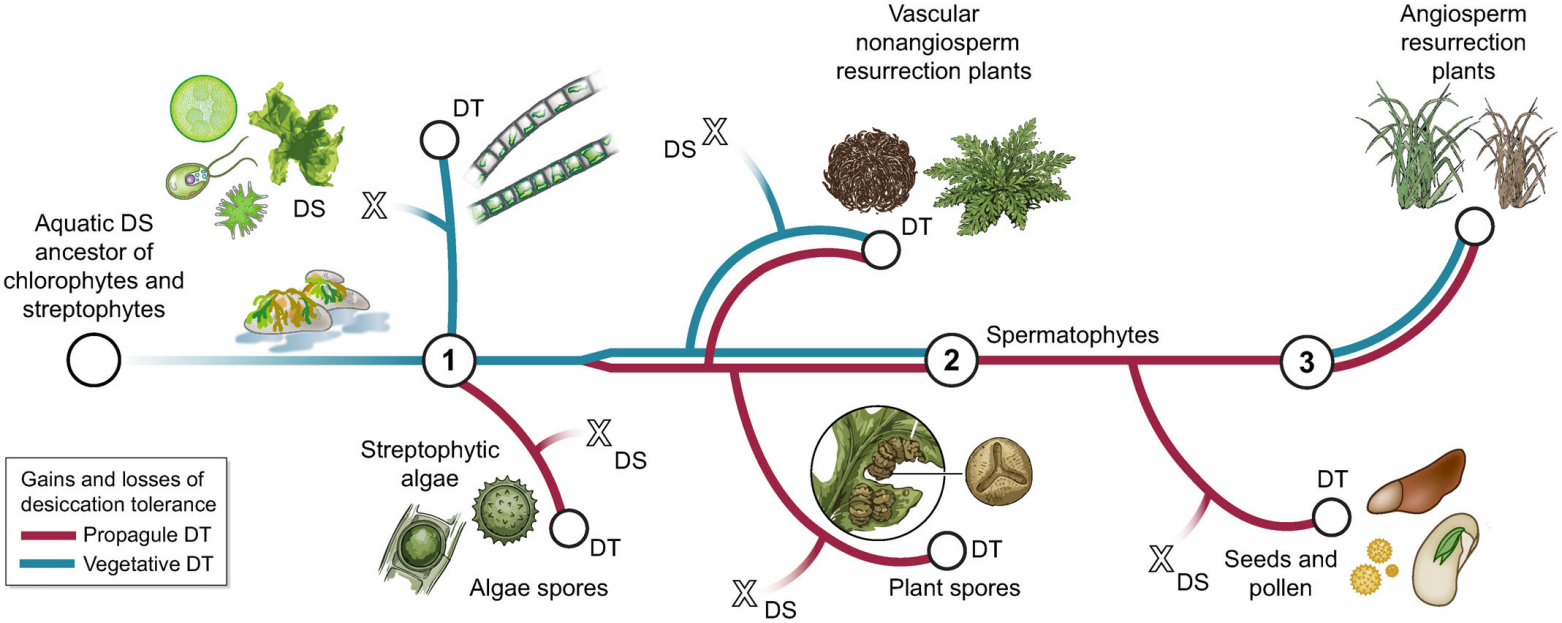
Ancient origin and recurrent evolution of desiccation tolerance in the green lineage. An aquatic desiccation‐sensitive ancestor gave rise to chlorophytes and streptophytes. (1) During early streptophyte evolution, desiccation tolerance evolved in propagules, such as algal spores and plant spores and was likely present in some vegetative tissues during terrestrialization. (2) As vascular plants diversified, vegetative desiccation tolerance was largely lost in most lineages when improved water transport and water use strategies evolved, with desiccation tolerance becoming primarily restricted to reproductive propagules, such as spores, seeds, and pollen. (3) In a small number of lineages, vegetative desiccation tolerance re‐evolved through the co‐option of propagule desiccation tolerance mechanisms, giving rise to resurrection plants in both non angiosperm and angiosperm clades. Colors indicate desiccation tolerance in vegetative tissues (blue) and propagules (red) and Xs denote loss of desiccation tolerance in sensitive lineages. DT, desiccation tolerant; DS, desiccation sensitive.

Early land plants were likely poikilohydrous, with limited ability to regulate internal water content independently of their surrounding environment. Fossil and phylogenetic evidence suggests that these plants were small and lacked many of the vascular, rooting, and water‐conserving features that evolved later (Rubinstein *et al*., 2010; de Vries & Archibald, 2018; Morris *et al*., 2018; Fig. 1). However, as vascular tissues, stomata, and other water‐conserving innovations evolved, desiccation tolerance was lost from most vegetative tissues in favor of mechanisms that maintain internal water balance. These underlying desiccation tolerance mechanisms were retained in most spores, seeds, and pollen, where they continue to support stress resistance, dormancy, dispersal, and long‐term survival through dry conditions (Oliver *et al*., 2000; Alpert, 2005; Gaff & Oliver, 2013). More recently, these seed pathways were likely co‐opted and rewired in angiosperm resurrection plants to enable vegetative desiccation tolerance, as supported by comparative genomic and transcriptomic studies (Cushman & Oliver, 2011; VanBuren *et al*., 2017; Costa *et al*., 2017b; Pardo *et al*., 2020). Consistent with this view, many of the gene families and stress‐response pathways underlying desiccation tolerance are conserved in streptophyte and chlorophyte algae, suggesting that the molecular toolkit for surviving drying predates the terrestrialization of plants (Rippin *et al*., 2017; Azar *et al*., 2025; Dadras *et al*., 2025). Together, these evolutionary patterns support the emerging view that desiccation tolerance is built from deeply conserved mechanisms, shaped by lineage‐specific rewiring rather than *de novo* innovation.

In this review, we examine the underlying mechanisms that have enabled the evolution of desiccation tolerance across the green lineage and beyond. We explore how this trait has emerged repeatedly through the recruitment of a shared molecular toolkit across vast evolutionary distances to stabilize cellular structures during extreme dehydration. We discuss hypotheses on the origin of desiccation tolerance and how ancient protective pathways have been recurrently repurposed to enable life without water. We also highlight the unique challenges that desiccation poses for photosynthetic organisms, and how plants, algae, and cyanobacteria mitigate the damaging effects of light and photooxidative stress in a dry state. Finally, we examine the emergent complexity of desiccation tolerance revealed through cross‐scale, systems‐level datasets, and discuss how this knowledge base may help us understand and inform efforts to engineer stress tolerance in crops and biotechnological systems.

## II. Core mechanisms underlying desiccation tolerance

Across the tree of life, organisms face the same fundamental cellular stressors during extreme dehydration and desiccation. This includes mechanical and biochemical stress, protein instability, aggregation and denaturation, loss of membrane fluidity, and massive reduction in cell volume. Unwanted interactions occur as cellular structure is dismantled, and proteins and macromolecules are brought into proximity with each other (Oliver *et al*., 2020). Desiccation‐tolerant organisms have evolved diverse mechanisms to stabilize macromolecules, preserve cellular integrity, and minimize damaging interactions. Many of these protective functions are shared across desiccation‐tolerant plants, invertebrates, fungi, and bacteria, consistent with a combination of deeply conserved stress‐response mechanisms, convergent evolution, and lineage‐specific innovation. Rather than implying a single evolutionary origin for desiccation tolerance across all lineages, these similarities suggest that desiccation tolerance has repeatedly drawn on ancient cellular stress‐resilience pathways and modified them in different evolutionary contexts.

In addition to these core cellular challenges, photosynthetic desiccation‐tolerant organisms must mitigate photooxidative damage driven by high irradiance and ultraviolet radiation. Plants, green algae, and cyanobacteria address these stressors using specialized photoprotective mechanisms that dissipate excess excitation energy and limit reactive oxygen species (ROS) formation. However, the extent, diversity, and functional importance of light‐induced damage and photoprotection during desiccation remain understudied.

### 1. Physical stabilization through vitrification and sugar accumulation

**Fig. 2:**
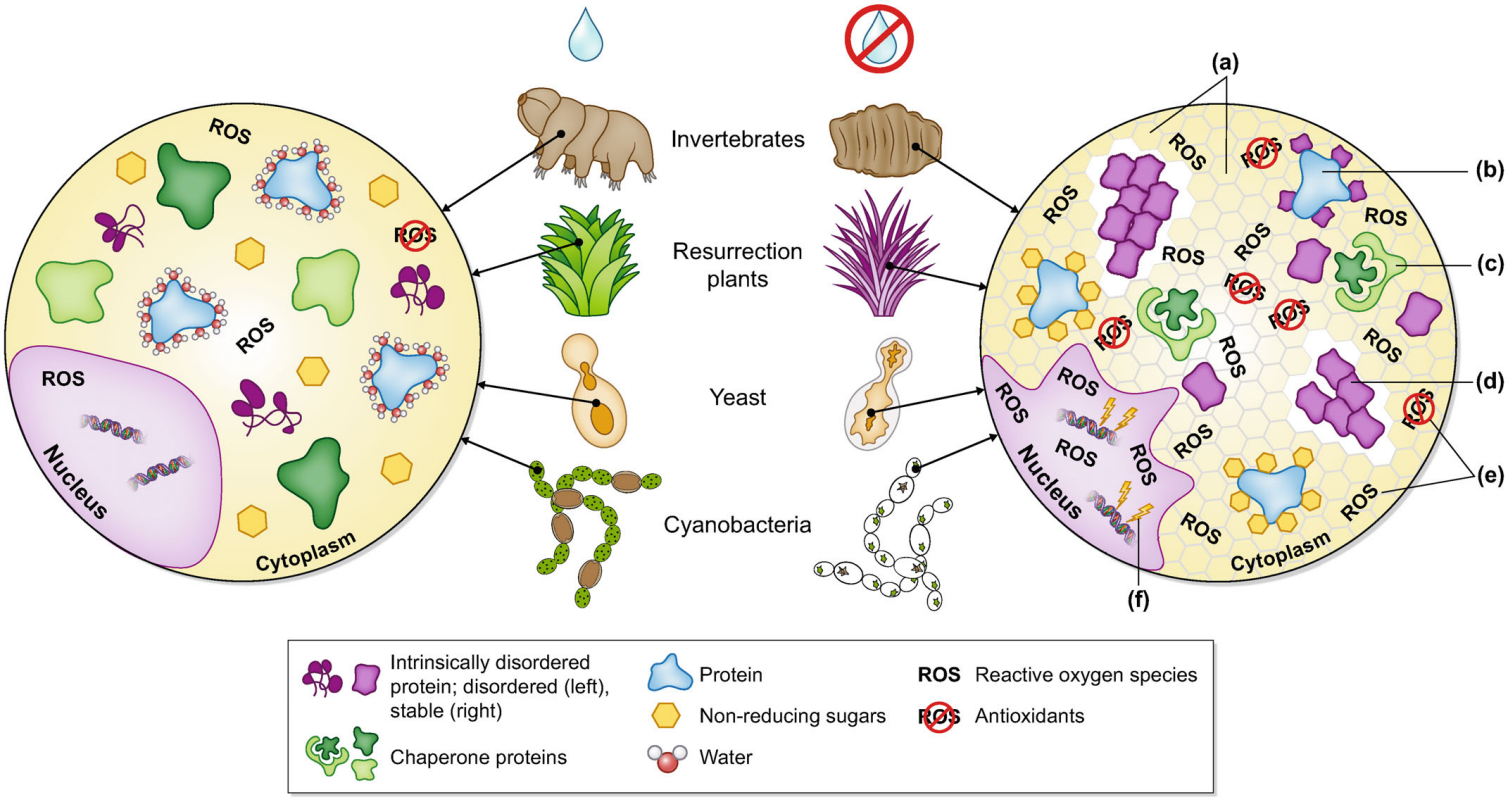
Shared cellular mechanisms underlying desiccation tolerance across the tree of life. Desiccation‐tolerant organisms, including cyanobacteria, yeast, invertebrates, and resurrection plants use shared biochemical and biophysical mechanisms to survive extreme cellular dehydration. These mechanisms stabilize macromolecules, protect cellular structures, and mitigate oxidative stress during water loss. (a) Nonreducing sugars accumulate and vitrify the cytoplasm. (b) Nonreducing sugars and intrinsically disordered proteins (IDPs) form hydrogen bonds with membranes and proteins, replacing water and maintaining protein structure and membrane stability. (c) Chaperone proteins prevent protein denaturation and aggregation. (d) IDPs stabilize at low water contents and contribute to vitrification. (e) Antioxidants accumulate to manage overproduction of reactive oxygen species (ROS). (f) DNA damage can occur due to ROS accumulation or prolonged exposure to high light in the desiccated state.

Nonreducing sugars accumulate in nearly all desiccation‐tolerant organisms during drying (Hoekstra *et al*., 2001; Reina‐Bueno *et al*., 2012; Tapia *et al*., 2015; Nguyen *et al*., 2022). Nonreducing sugars are chemically stable and minimally reactive, which makes them less prone to degradation or unwanted reactions during desiccation. Accumulation of nonreducing sugars, such as sucrose and trehalose, promotes the transition of the cytoplasm from a gel‐like state to a glassy, vitrified matrix (Fig. 2; Crowe *et al*., 1998a; Buitink & Leprince, 2004). Vitrification, or the formation of a biological glass, stabilizes the cellular environment by immobilizing macromolecules and cellular components, relieving mechanical stress on membranes, and limiting undesirable molecular interactions caused by cell shrinkage and cytoplasmic crowding. Nonreducing sugars have high glass transition temperatures, the temperature range at which a rigid, glassy structure becomes more viscous (Wolkers *et al*., 1998; Crowe *et al*., 1998a). This enables biological glasses to preserve cellular structures for extended periods in the dry state. Additionally, nonreducing sugars are hydrophilic and can form hydrogen bonds with proteins and polar head groups of phospholipid membranes. As water is lost during drying, nonreducing sugars can functionally replace water by forming similar hydrogen bonds, preventing the denaturation of proteins and maintaining membrane fluidity during drying (Fig. 2; Clegg *et al*., 1982; Crowe *et al*., 1998b).

Sucrose is the most prominent nonreducing sugar accumulated during drying in most resurrection plants (Dace *et al*., 2023). In some species, sucrose can accumulate to 90% of dry weight in desiccated tissues (Bianchi *et al*., 1991; Whittaker *et al*., 2001; Zhang *et al*., 2016). This accumulation results from both *de novo* synthesis and starch degradation, based on increased activity of enzymes in the sucrose biosynthetic pathway and elevated breakdown of starch (Müller *et al*., 1997; Whittaker *et al*., 2007). In *Craterostigma* and other Linderiaceae species, the unusual sugar octulose accumulates during drying and is later converted into sucrose, contributing to the sugar pool for vitrification (Phillips *et al*., 2008; Egert *et al*., 2015). Raffinose family oligosaccharides, such as raffinose and stachyose, also accumulate in desiccation‐tolerant seeds and leaves of resurrection plant species, such as *Xerophyta viscosa* and *Eragrostis nindensis*, and contribute to water replacement, vitrification, and protection from oxidative damage (Koster, 1991; Hincha *et al*., 2003; Nishizawa *et al*., 2008). Similarly, desiccation‐tolerant cyanobacteria produce extracellular polysaccharide structures that function similarly to nonreducing sugars, stabilizing the cells during desiccation (Wright *et al*., 2005).

Trehalose, another nonreducing disaccharide, has a well‐characterized role in desiccation tolerance in yeast, nematodes, and *Artemia*, and functions analogously to sucrose through stabilizing macromolecules in the dry state (Hibshman *et al*., 2020). Although trehalose is synthesized in some resurrection plants, including *Selaginella* species, *Myrothamnus flabellifolia*, and *Tripogon lolliformis*, its accumulation is lower than that of sucrose (Adams *et al*., 1990; Moore *et al*., 2007; Williams *et al*., 2015). Trehalose also varies across desiccation‐tolerant animals, as some tardigrades and rotifers accumulate very little trehalose, and some tardigrade species appear to lack canonical trehalose biosynthetic genes altogether (Boothby *et al*., 2017; Boothby & Pielak, 2017). However, low amounts of trehalose still contribute to desiccation tolerance in tardigrades by enhancing the function of cytoplasmic abundant heat soluble (CAHS) proteins that promote vitrification and cellular stabilization during drying (Nguyen *et al*., 2022). Despite differences in the identity and abundance of specific sugars, their shared role in promoting glass formation, stabilizing macromolecules, and replacing water highlights vitrification as a unifying mechanism of desiccation tolerance shaped by evolutionary constraints on maintaining cellular integrity in the absence of water.

### 2. Specialized protective proteins

In addition to sugars, desiccation‐tolerant organisms rely heavily on proteins that perform protective functions during drying. Among these, intrinsically disordered proteins (IDPs) play a particularly important role due to their structural flexibility and multifunctionality. IDPs lack a fixed three‐dimensional structure and participate in diverse cellular processes (Wright & Dyson, 2015). Desiccation tolerance IDPs (DT‐IDPs) have hydrophilic properties that allow them to perform many protective functions similar to those of sugars, including facilitating vitrification and replacing bound water (Fig. 2). Like sugars, DT‐IDPs are able to form hydrogen bonds with membranes and other proteins during drying, substituting for water molecules to maintain membrane fluidity and prevent protein denaturation (Sochava & Smirnova, 1993; Boothby & Pielak, 2017). In addition, DT‐IDPs are also involved in scavenging ROS and function as chaperone proteins (Tunnacliffe & Wise, 2007; Liu *et al*., 2023).

While DT‐IDPs can form protective biological glasses, they also increase the glass transition temperature of sugar‐based vitrified cytoplasm, thereby improving glass stability (Fig. 2; Elliott *et al*., 1996; Kim *et al*., 2018). Late embryogenesis abundant (LEA) proteins, which contribute to vitrification in most desiccation‐tolerant organisms, increase the glass transition temperature of sucrose‐based glasses in resurrection plants and desiccation‐tolerant seeds (Wolkers *et al*., 2001). Additionally, in tardigrades, CAHS proteins act together with trehalose to strengthen biological glasses (Nguyen *et al*., 2022).

Heat shock proteins (HSPs) are present in all eukaryotic organisms and play a crucial role in the stabilization of macromolecules during desiccation (Parsell & Lindquist, 1993; Richter *et al*., 2010; Hibshman *et al*., 2020). A family of HSPs known as small heat shock proteins (sHSPs) have intrinsically disordered regions, similar to LEA and CAHS proteins, and act as molecular chaperones to prevent the denaturation and aggregation of key proteins during desiccation and rehydration (Haslbeck & Vierling, 2015; Hibshman *et al*., 2023; Mihailova *et al*., 2023). sHSPs are expressed during the development of pollen and seeds and have been implicated in desiccation tolerance in vegetative plant tissues, as well as in other desiccation‐tolerant organisms like tardigrades, nematodes, and *Artemia* (Alamillo *et al*., 1995; Wehmeyer & Vierling, 2000; Hibshman *et al*., 2020). While HSP70, a canonical HSP is upregulated in response to dehydration in plants and desiccation‐tolerant invertebrates, its role appears to be a part of a general stress response rather than a mechanism specific for desiccation tolerance (Aghaie & Tafreshi, 2020; Hibshman *et al*., 2020).

**Fig. 3:**
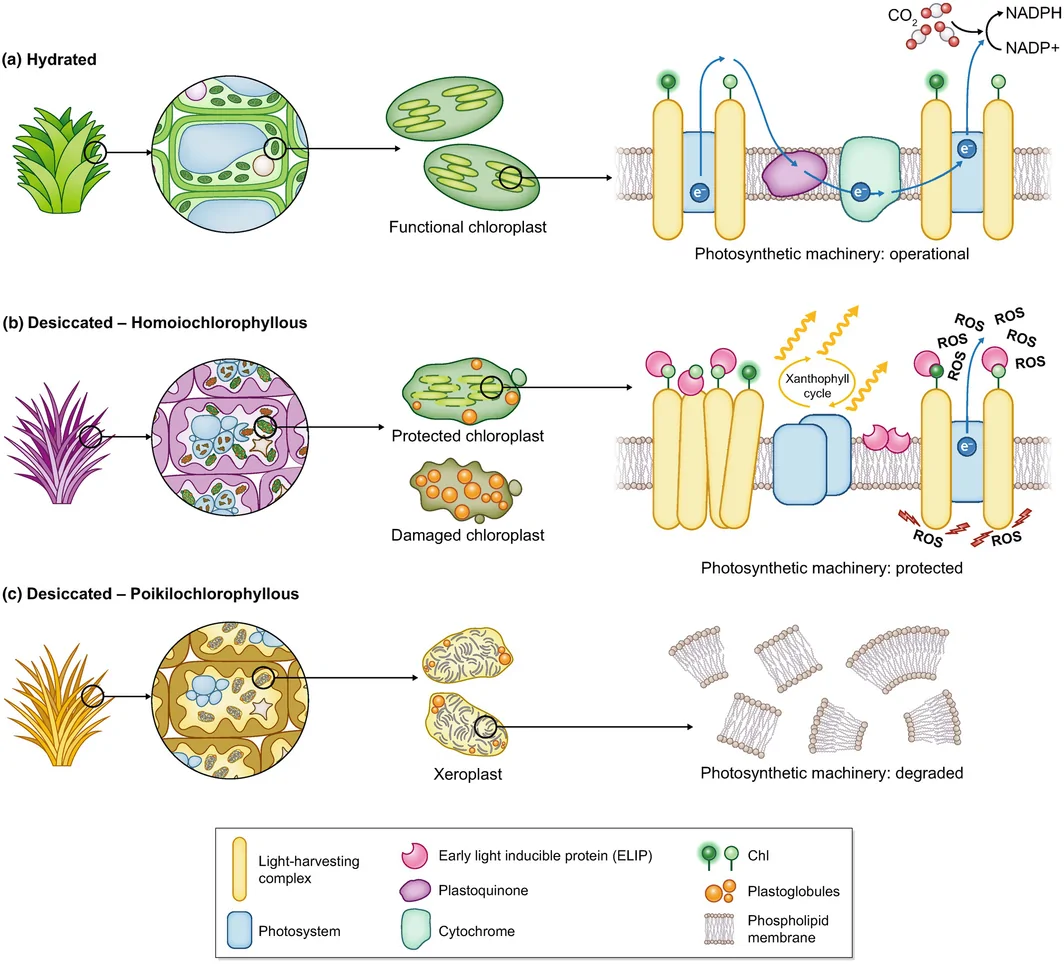
Photosynthetic adaptations to desiccation across organizational scales. Desiccation‐tolerant plants employ structural, cellular, and molecular strategies to manage light energy and protect photosynthetic machinery during dehydration. (a) Hydrated. In hydrated tissues, chloroplasts are functional and the photosynthetic apparatus is operational. Light‐harvesting complexes and photosystems drive electron transport, producing ATP and NADPH that fuel carbon fixation in the Calvin Cycle when CO_2_ is available. (b) Desiccated, homoiochlorophyllous species. Chl and photosynthetic structures are retained but protected during dehydration. During drying, stomatal closure and declining water content limit CO_2_ uptake and carbon fixation, creating redox imbalance while light absorption continues. As tissues enter the dry state, photosynthesis and electron transport are largely inactive, but retained pigments and photosynthetic complexes remain vulnerable to light‐dependent oxidative damage. Stomata close and CO_2_ becomes limiting, halting the Calvin Cycle. LHCs detach from photosystems, ELIPs bind Chl and absorb excess energy, and the xanthophyll cycle dissipates excitation energy as heat during drying and early recovery. Plastoglobules accumulate in chloroplasts and damaged organelles may be recycled through autophagy during drying and rehydration, while protected chloroplasts remain intact for rapid recovery upon rehydration. (c) Desiccated, poikilochlorophyllous species. Photosynthetic structures are largely dismantled during drying. Thylakoid membranes, Chl, and photosystems are degraded, leaving lipid remnants within xeroplasts. The photosynthetic machinery is subsequently rebuilt during rehydration.

Resurrection plants also rely on specialized proteins to protect against oxidative and structural damage caused by exposure to intense light. Early light‐inducible proteins (ELIPs) in resurrection plants and related high light‐inducible proteins (HLIPs) in desiccation‐tolerant terrestrial cyanobacteria belong to the broader light‐harvesting‐complex‐like (LHC‐like) protein superfamily, a group of pigment‐binding proteins that help protect photosynthetic machinery from photooxidative damage (Fig. 3; Hutin *et al*., 2003). Green algae and land plants also use members of the LHCSR and PsbS protein families to dissipate excess excitation energy under high light (Peers *et al*., 2009; Tibiletti *et al*., 2016; Xu *et al*., 2022). In addition, *Craterostigma plantagineum* expresses a plastid‐localized DNA‐binding protein (CpPTP) that translocates to the chloroplast. CpPTP has been proposed to downregulate chloroplast activity during drying (Phillips *et al*., 2002; Dinakar & Bartels, 2012).

Protein‐based protection is a central component of survival in the dry state. However, many of these DT‐IDPs and other protective proteins are also induced in desiccation‐sensitive species during drying, including in LEAs in recalcitrant seeds (Pammenter & Berjak, 1999) and vegetative tissues of sensitive relatives of resurrection plants (VanBuren *et al*., 2018a; Pardo *et al*., 2020). Instead, desiccation tolerance appears to depend on the coordination, timing, and magnitude of these responses.

### 3. Mitigating oxidative stress

In addition to structural and biochemical challenges imposed by water loss, desiccating cells experience severe oxidative stress throughout drying, in the dry state, and during rehydration, although the sources and consequences of ROS differ across these phases. ROS can damage membranes, nucleic acids, and proteins and arise from electron leakage in mitochondrial and chloroplast electron transport chains. During drying, declining water content progressively restricts carbon fixation and photosynthetic electron transport, creating redox imbalance. In fully desiccated tissues, photosynthesis is inactive, but retained pigments and photosynthetic complexes can remain vulnerable to light‐dependent oxidative damage. ROS production can also increase during recovery as respiration and photosynthesis resume. Thus, photoprotection is a critical component of desiccation tolerance in photosynthetic organisms and requires specialized structural, biochemical, and regulatory strategies not needed in most nonphotosynthetic taxa.

Resurrection plants have evolved two major strategies for reducing photooxidative stress during drying. Homoiochlorophyllous species retain and protect Chl and thylakoid membranes throughout desiccation, which allows faster recovery of photosynthesis but increases the risk of light‐dependent oxidative damage while tissues are dry (Fig. 3; Tuba *et al*., 1998; Dinakar & Bartels, 2012; Charuvi *et al*., 2015, 2019; Oliver *et al*., 2020). By contrast, poikilochlorophyllous species dismantle Chl and photosynthetic structures during drying and rebuild them after rehydration, reducing photooxidative risk in the dry state but delaying photosynthetic recovery. Most resurrection plants are homoiochlorophyllous, and this strategy is advantageous in habitats with short, unpredictable wet periods (Tuba & Lichtenthaler, 2011).

Although homoiochlorophylly makes recovery from desiccation much quicker, it also makes plants more susceptible to oxidative stress. To counteract this, these species may employ both physical shielding mechanisms and biochemical reorganization of their photosystems (Tuba & Lichtenthaler, 2011; Zia *et al*., 2016). Some species physically protect themselves by folding or curling their leaves to shade them from direct sunlight (Schwab *et al*., 1989; Barták *et al*., 2006; Georgieva *et al*., 2009; Rafsanjani *et al*., 2015). *Myrothamnus flabellifolius* is unable to recover when leaf folding is restricted, suggesting this physical avoidance mechanism may be required for recovery in some resurrection plants (Farrant *et al*., 2003). In *Craterostigma wilmsii*, thylakoids migrate to one side of the chloroplast during dehydration, likely providing an additional layer of protection against direct light alongside leaf folding (Cooper & Farrant, 2002; Costa *et al*., 2017b). Under desiccation, light‐harvesting complexes (LHCII) detach from photosystem II (PSII) and PSIIs aggregate into semi‐crystalline arrays, which halts the light‐dependent reactions, causing light energy captured by LHCII to be dissipated via carotenoids in a form of nonphotochemical quenching (Fig. 3; Charuvi *et al*., 2015; Zia *et al*., 2016). Similarly, Chlamydomonas and other green algae undergo state transitions under high light, where LHCII shifts from PSII to PSI to rebalance electron flow (Bellafiore *et al*., 2005). Because desiccation also over‐reduces the photosynthetic chain, similar transitions may provide photoprotection in desiccation‐tolerant algae and possibly chlorolichens (Gauslaa *et al*., 2012).

Anthocyanins and carotenoids complement the physical avoidance mechanisms of homoiochlorophyllous resurrection plants, and they accumulate to high levels on the outermost layers of leaves during drying (Fig. 3; Sherwin & Farrant, 1998; Farrant *et al*., 2003; Charuvi *et al*., 2019). These pigments absorb excess light and reduce Chl excitation, with anthocyanins also acting as antioxidants. The accumulation of these pigments helps limit Chl overexcitation, reduce ROS accumulation, and protect leaves from direct sunlight, which is especially important in homoiochlorophyllous species that retain photosynthetic machinery during drying (Sherwin & Farrant, 1998). Cyanobacteria utilize specialized protective molecules, like scytonemin and mycosporine‐like amino acids, which act similarly to anthocyanins in plants by protecting against harmful UV radiation (Singh *et al*., 2010; Rastogi *et al*., 2015). Although these compounds are primarily induced by UV exposure, they are important in extreme environments where high irradiance, UV exposure, and periodic drying co‐occur (Fleming & Castenholz, 2007). Similarly, cyanobacterial photobionts in some lichens have been shown to produce scytonemin and other photoprotectants (Nguyen *et al*., 2013).

While homoiochlorophyllous species invest heavily in retaining and protecting their photosynthetic machinery, other desiccation‐tolerant plants take the opposite approach. Poikilochlorophyllous species dismantle their photosynthetic apparatus during drying and rebuild it after rehydration, thereby minimizing the risk of light‐induced damage while desiccated (Fig. 3; Gaff, 1977; Vander Willigen *et al*., 2001). This prevents ROS accumulation from Chl overexcitation, but it results in a slower recovery, as the photosynthetic machinery must be reassembled upon rehydration (Vander Willigen *et al*., 2001). In angiosperms, poikilochlorophylly is observed only in monocots and is typically associated with species adapted to hyper‐arid environments or high‐light savannahs, where retaining and protecting the photosynthetic machinery during desiccation would be too costly (Proctor & Tuba, 2002). These divergent strategies reflect ecological trade‐offs and distinct photoprotective innovations that plants must evolve to survive desiccation in the presence of light.

Poikilochlorophylly depends on coordinated Chl catabolism, transient photoprotection, and stored transcripts that allow the photosynthetic apparatus to be dismantled during drying and rebuilt after rehydration. Chl degradation in resurrection plants was initially thought to be nonenzymatic (Proctor & Tuba, 2002). However, later work showed that poikilochlorophyllous plants degrade their Chl using the pheophorbide α oxygenase (PAO)/phyllobin pathway, a process that mirrors Chl breakdown in senescent tissues (Christ *et al*., 2014). Anthocyanins accumulate during the early stages of rehydration, protecting the cell from ROS generation as the thylakoids and photosynthetic apparatus are reassembled (Sherwin & Farrant, 1998). To ensure a rapid recovery, poikilochlorophyllous species accumulate the transcripts necessary to reassemble the photosynthetic apparatus quickly upon rehydration (Dace *et al*., 1998). This strategy mirrors regulatory processes seen during seed maturation, where Chl breakdown and mRNA storage prepare cells for desiccation and later reactivation. These parallels suggest that vegetative desiccation tolerance in poikilochlorophyllous plants may co‐opt regulatory and catabolic programs originally evolved for seed desiccation (Cooper & Farrant, 2002; Costa *et al*., 2017b).

Even when photoprotective mechanisms reduce light‐induced damage, ROS can still accumulate during drying, dry storage, and rehydration, making antioxidant defenses essential for desiccation tolerance. Across the tree of life, desiccation‐tolerant species rely on conserved antioxidant systems to mitigate ROS damage during dehydration and rehydration (Fig. 2), including nonenzymatic antioxidants, such as glutathione, carotenoids, ascorbic acid, and tocopherols, and enzymes, such as superoxide dismutase (SOD), catalase, and glutathione reductase. These antioxidant systems are found in diverse desiccation‐tolerant species, including resurrection plants, seeds, tardigrades, nematodes, and midge larvae (Adhikari *et al*., 2009; Moore *et al*., 2009; Rizzo *et al*., 2010; Tyson *et al*., 2012; Nesmelov *et al*., 2018; Giovannini *et al*., 2022). For example, in the resurrection plant *Haberlea rhodopensis*, ascorbic acid increases fivefold and glutathione increases 50‐fold during drying (Georgieva *et al*., 2017). Antioxidants such as 1‐ and 2‐cys‐peroxiredoxins, glyoxalase I family proteins, and zinc metallothionine are found in resurrection plants and desiccation‐tolerant seeds but not in desiccation‐sensitive species. Additionally, SOD and glutathione reductase activity increase during drying in two tardigrade species (Rizzo *et al*., 2010; Giovannini *et al*., 2022). While many antioxidants are not exclusive to desiccation‐tolerant organisms, the high concentration and preservation of these molecules in the dry state are hallmarks of desiccation tolerance (Kranner & Birtic, 2005). Even at low hydration levels in the glassy state, some respiration and ROS production continue, but antioxidant capacity persists, mitigating this damage (Farrant, 2000; Kranner *et al*., 2002).

### 4. Cellular recycling and organelle repair

Despite the protective mechanisms described above, some cellular damage during desiccation is unavoidable, especially across repeated dehydration and rehydration cycles. Desiccation‐tolerant organisms activate autophagy during drying and recovery to recycle damaged proteins, membranes, and organelles. Autophagy‐related processes are upregulated in resurrection plants, yeast, Artemia, and the sleeping chironomid, while programmed cell death pathways are reduced or suppressed (Ratnakumar *et al*., 2011; Lin *et al*., 2016; Belott *et al*., 2024).

In photosynthetic organisms, autophagy may help recycle photosynthetic machinery that is damaged during drying or from prolonged light exposure in the desiccated state Fig. 3. Unlike seeds, which dismantle chloroplasts during maturation and rebuild them during germination, or senescing leaves, which dismantle chloroplasts irreversibly, poikilochlorophyllous resurrection plants disassemble, and rapidly reassemble photosynthetic structures during desiccation and recovery over multiple drying/rehydration cycles (Ooms *et al*., 1993; Pogson & Albrecht, 2011). In the homoiochlorophyllous eudicot *Craterostigma pumilum*, desiccation leads to an accumulation of plastoglobules, chloroplast fragmentation, and selective vacuolar degradation of damaged thylakoid membranes, with some membrane fragments sequestered within plastoglobules. Thus, although homoiochlorophyllous species retain much of their photosynthetic machinery during drying, autophagy and vacuolar degradation may help remove damaged photosynthetic components during desiccation and recovery (Charuvi *et al*., 2019; Fig. 3).

Xeroplasts, the desiccated chloroplasts of poikilochlorophyllous species, resemble gerontoplasts, degraded, nonphotosynthetic chloroplasts in senescent tissues. Rather than being degraded, they are reactivated and differentiate back into functional chloroplasts upon rehydration (Ingle *et al*., 2008). Plastoglobules have also been implicated in thylakoid membrane assembly during biogenesis and disassembly during senescence (Rottet *et al*., 2015). Fractured thylakoid membranes might be repaired or recycled upon rehydration via co‐option of these biogenesis or autophagy pathways in poikilochlorophyllous species (Besagni & Kessler, 2013; Rottet *et al*., 2015; Charuvi *et al*., 2019).

### 5. DNA damage and repair

DNA damage poses a major threat to genome integrity during desiccation, largely due to oxidative stress and UV exposure during both drying and recovery. Although antioxidants and ROS‐scavenging enzymes protect cellular components, they cannot fully prevent DNA damage (Waterworth *et al*., 2019). Most desiccation‐associated DNA damage appears to arise indirectly through oxidative stress and dry‐state exposure, making rapid repair essential during recovery (Gladyshev & Meselson, 2008; Neumann *et al*., 2009; Gladyshev & Arkhipova, 2010; Gusev *et al*., 2010; Rebecchi, 2013). The extreme radiation tolerance of many desiccation‐tolerant organisms, including rotifers, Artemia, chironomid larvae, tardigrades, and seeds, is linked to efficient DNA protection and repair systems (Gladyshev & Meselson, 2008; Neumann *et al*., 2009; Gusev *et al*., 2010; Marcu *et al*., 2013; MacRae, 2016). These mechanisms include conserved pathways, such as base excision repair, nucleotide excision repair, and double‐strand break repair, which are active during rehydration (Waterworth *et al*., 2019). For example, RAD51‐mediated double‐strand break repair supports recovery from desiccation‐related stress across species, with rehydration‐induced expression in *Polypedilum vanderplanki* and reduced germination and survival in maize mutants (Li *et al*., 2007; Gusev *et al*., 2010). Oxidative lesions such as 8‐oxoguanine also accumulate in desiccating seeds and are repaired by OGG1 and FPG during imbibition, while additional repair pathways such as nucleotide excision repair and DNA ligase activity contribute to seed longevity and genome restoration during germination (Macovei *et al*., 2011; Chen *et al*., 2012; Parreira *et al*., 2018).

In addition to repair, some proteins provide direct protection to DNA. Group II LEA proteins can bind DNA and may contribute to DNA protection, repair, or chromatin‐associated processes during stress (Wise & Tunnacliffe, 2004; Banerjee & Roychoudhury, 2016). Furthermore, the Damage Suppressor (Dsup) protein, specific to tardigrades, binds to DNA and protects it from radiation induced damage (Hashimoto *et al*., 2016). Dsup has been shown to reduce radiation damage in human cells and plants, and could safeguard DNA from ROS associated damage during desiccation (Hashimoto *et al*., 2016; Kirke *et al*., 2020; Del Casino *et al*., 2024). Given the repeated evolution of desiccation tolerance and shared strategies across taxa, it is plausible that functionally similar DNA‐protective proteins exist in other desiccation‐tolerant organisms, although this is understudied. Together, these proteins complement enzymatic repair pathways and contribute to the broader network of mechanisms that preserve genome integrity during extreme dehydration.

## III. The emergent complexity of desiccation tolerance in plants

Desiccation tolerance is an emergent trait that arises from numerous molecular, physiological, and cellular processes coordinated across time and space that give rise to a phenotype that cannot be predicted from single pathways in isolation. Understanding this complexity has only become possible through advances in genome‐scale and systems‐level approaches, together with a growing research community, which have brought the molecular mechanisms underlying desiccation tolerance into clearer focus (Gechev *et al*., 2021; Tebele *et al*., 2021; Lotreck *et al*., 2024). As of mid‐2025, > 20 high‐quality reference genomes and dozens of multi‐omics studies have been published, profiling gene expression (transcriptomics), metabolite profiles (metabolomics), protein accumulation and modification (proteomics), and chromatin and methylation dynamics (epigenomics) during desiccation and rehydration (Gechev *et al*., 2021; Gao *et al*., 2024). Collectively, these large‐scale datasets have revealed that desiccation tolerance is an unexpectedly complex trait, involving far more regulatory and metabolic pathways than early single‐gene studies suggested. Tolerance is orchestrated through coordinated changes across time and tissues at multiple biological levels. Comparative datasets across resurrection plants have demonstrated extensive network rewiring, most notably the co‐option of seed developmental programs into vegetative tissues (VanBuren *et al*., 2017, 2018a; Pardo *et al*., 2020). Shared molecular, physiological, biochemical, and genomic signatures further support the existence of core mechanisms underlying this trait. Yet despite these advances, many aspects of desiccation tolerance still remain poorly understood, and its complexity continues to challenge our ability to model, predict, engineer, or fully reconstruct this phenotype.

Before the 1980s, research on resurrection plants focused largely on physiology and morphology, and the broader plant biology community was mostly unaware of these species (Bartels *et al*., 2025). The molecular era began in 1990, when Dorothea Bartels *et al*. (1990) first investigated desiccation tolerance at the gene level in the resurrection plant *Craterostigma plantagineum*. Using abscisic acid (ABA) pretreatment and differential cDNA screening, they identified the first desiccation‐inducible genes, many encoding LEA proteins, linking vegetative desiccation tolerance to seed dehydration pathways. This work catalyzed molecular studies of tolerance, revealing numerous protective genes, including dehydrins, ELIPs, antioxidants, and stress‐responsive transcription factors. Early transcriptomic, proteomic, and metabolomic studies later laid the groundwork for modern systems‐level analyses (Ingle *et al*., 2007; Jiang *et al*., 2007; Rodriguez *et al*., 2010).

### 1. Genome architecture and desiccation tolerance

Genomic resources for resurrection plants initially lagged behind those of model systems, crops, and economically relevant plants (Marks *et al*., 2021b). This changed with the publication of the high‐quality genome for the resurrection grass *Oropetium thomaeum*, which provided new insights into the genetic basis of desiccation tolerance (VanBuren *et al*., 2015, 2018b). Since then, genomes have been sequenced for numerous additional resurrection species (Table 1). These new genomic resources have enabled the community to test longstanding hypotheses about the genetic basis of desiccation tolerance, including potential links to polyploidy (Marks *et al*., 2024a), the presence of desiccation‐associated gene islands (Gusev *et al*., 2014; Costa *et al*., 2017a), shifts in genome architecture, and the repurposing of ancestral seed‐related pathways (Gaff & Oliver, 2013).

**Table 1:** Overview of sequenced resurrection plants.

Species	Clade	Family	Genome size	References	Ploidy	Desiccation tolerance strategy
*Selaginella lepidophylla*	Lycophyte	Selaginellaceae	109 Mb	VanBuren *et al*. (2018c)	Diploid	Homoiochlorophyllous
*Selaginella tamariscina*	Lycophyte	Selaginellaceae	301 Mb	Xu *et al*. (2018)	Diploid	Homoiochlorophyllous
*Selaginella sellowii*	Lycophyte	Selaginellaceae	74.06 Mb	Alejo‐Jacuinde *et al*. (2025)	Diploid	Homoiochlorophyllous
*Syntrichia caninervis*	Bryophyte	Pottiaceae	292.2 Mb	Silva *et al*. (2021)	Diploid	Homoiochlorophyllous
*Syntrichia ruralis*	Bryophyte	Pottiaceae	305.19 Mb	Zhang *et al*. (2024)	Diploid	Homoiochlorophyllous
*Pohlia nutans*	Bryophyte	Mniaceae	698.2 Mb	Liu *et al*. (2022)	Diploid	Homoiochlorophyllous
*Ceratodon purpureus*	Bryophyte	Ditrichaceae	358 Mb	Carey *et al*. (2020)	Diploid	Homoiochlorophyllous
*Fontinalis antipyretica*	Bryophyte	Fontinalaceae	385.2 Mb	Yu *et al*. (2020)	Diploid	Homoiochlorophyllous
*Boea hygrometrica*	Angiosperm	Gesneriaceae	1691 Mb	Xiao *et al*. (2015)	Diploid	Homoiochlorophyllous
*Craterostigma plantagineum*	Angiosperm	Linderniaceae	1030 Mb	VanBuren *et al*. (2023)	Octoploid	Homoiochlorophyllous
*Lindernia brevidens*	Angiosperm	Linderniaceae	270 Mb	VanBuren *et al*. (2018a)	Diploid	Homoiochlorophyllous
*Myrothamnus flabellifolia*	Angiosperm	Myrothamnaceae	1283 Mb	Marks *et al*. (2025b)	Diploid	Homoiochlorophyllous
*Haberlea rhodopensis*	Angiosperm	Gesneriaceae	1270 Mb	Gupta *et al*. (2024)	Diploid	Homoiochlorophyllous
*Acanthochlamys bracteata*	Angiosperm	Velloziaceae	197.97 Mb	Gao *et al*. (2021)	Diploid	Homoiochlorophyllous
*Xerophyta schlechteri*	Angiosperm	Velloziaceae	295.5 Mb	Costa *et al*. (2017a)	Octoploid	Poikilochlorophyllous
*Sporobolus stapfianus*	Angiosperm	Poaceae	1079 Mb	Montes *et al*. (2022)	Tetraploid	Homoiochlorophyllous
*Microchloa afra*	Angiosperm	Poaceae	968 Mb	Marks *et al*. (2024b)	Hexaploid	Homoiochlorophyllous
*Oropetium capense*	Angiosperm	Poaceae	237 Mb	Marks *et al*. (2024b)	Diploid	Homoiochlorophyllous
*Tripogon minimus*	Angiosperm	Poaceae	233 Mb	Marks *et al*. (2024b)	Diploid	Homoiochlorophyllous
*Eragrostis nindensis*	Angiosperm	Poaceae	986 Mb	Pardo *et al*. (2020)	Hexaploid	Poikilochlorophyllous
*Oropetium thomaeum*	Angiosperm	Poaceae	244 Mb	VanBuren *et al*. (2015, 2018b)	Diploid	Homoiochlorophyllous

One hypothesis proposes that polyploidy may have facilitated the evolution of desiccation tolerance, given its frequent association with increased environmental resilience in plants (Lohaus & Van de Peer, 2016). Several resurrection grasses, such as *Eragrostis nindensis*, *Sporobolus stapfianus*, and *Microchloa afra*, are complex polyploids. However, current evidence does not indicate that polyploidy is a defining feature of resurrection plants. Many related resurrection grasses, such as *Oropetium thomaeum*, *O. capense*, and various *Tripogon* species, are diploid with small *c*. 250‐Mb genomes (VanBuren *et al*., 2015; Marks *et al*., 2024b). Polyploidy is extremely common in grasses, and *c*. 90% of Chloridoid grasses are polyploid, regardless of tolerance (Roodt & Spies, 2003). While not essential for evolving tolerance, polyploidy may enhance it, as *Microchloa afra* genotypes with higher order ploidy are more desiccation‐tolerant (Marks *et al*., 2024a). Similarly, genome size has been proposed as a factor for desiccation tolerance, as many resurrection plants have relatively small monoploid genomes. Yet this pattern is inconsistent as several angiosperm resurrection plants and nearly all tolerant ferns have large, multi‐gigabase genomes. Thus, genome architectures may facilitate or enhance desiccation tolerance in some lineages, but neither polyploidy nor genome size currently appears to be a universal predictor of the trait.

Desiccation‐tolerant animals like *Polypedilum vanderplanki* have evolved desiccation‐associated gene islands, clusters of paralogous genes encoding protective proteins that are highly expressed during drying (Gusev *et al*., 2014). Similar clusters of desiccation‐associated genes were identified in the resurrection plant *Xerophyta schlecteri* (Costa *et al*., 2017a), but these patterns are difficult to interpret as defining features of desiccation‐tolerant plant genomes, as *c*. 20% of plant genes are tandemly duplicated, and some will inevitably be related to stress responses. Thus, neither polyploidy nor changes in genome architecture are defining features of resurrection plants.

One notable exception to otherwise typical plant genome architecture in resurrection plants is the dramatic expansion of ELIP gene families. ELIPs are small photoprotective proteins that help safeguard the photosynthetic machinery during light and dehydration stress (Hutin *et al*., 2003). Most land plants have one to five ELIPs, but every sequenced resurrection plant genome harbors dozens to hundreds, often in large tandem arrays with strong upregulation during desiccation and rehydration (VanBuren *et al*., 2019; Marks *et al*., 2024b). In angiosperms, these tandem duplicates are highly similar in sequence, consistent with recent lineage‐specific expansions, whereas in nonseed plants, such as bryophytes and lycophytes, ELIPs are more divergent and appear to have arisen from more ancient duplications (VanBuren *et al*., 2018c; Silva *et al*., 2021; Zhang *et al*., 2024; Alejo‐Jacuinde *et al*., 2025). Within grasses, ELIP duplication occurred independently in multiple Chloridoideae lineages, with Tripogoninae species showing tandem expansions on Chromosome 8 and Eleusininae and Eragrostidinae on Chromosome 7 (Marks *et al*., 2024b). Massive ELIP expansions are also observed in desiccation‐tolerant streptophytic algae, including within Zygnematophyceae, the closest algal relatives of land plants (Feng *et al*., 2024). Strikingly, duplication of ELIP homologs, the HLIPs, also occurs in desiccation‐tolerant cyanobacteria. In the desert cyanobacterium *Nostoc flagelliforme*, four tandemly repeated HLIPS confer protection during water deficit, and heterologous expression enhances desiccation tolerance in sensitive strains (Xu *et al*., 2022). ELIP and HLIP tandem duplications are a striking example of convergent evolution, representing independent expansions of the same gene family in response to similar selective pressures across cyanobacteria, algae, and land plants.

Taken together, these findings indicate that features, such as polyploidy, genome size, and large‐scale architectural changes, have had limited roles in the evolution of desiccation tolerance. Instead, this trait likely evolved through the rewiring and redeployment of existing genes and regulatory networks.

### 2. Recurrent evolution through regulatory reprogramming

In resurrection plants, time course transcriptomes show dramatic shifts in gene expression, with thousands of coordinated changes required to protect cellular macromolecules during drying and activate repair mechanisms upon rehydration (VanBuren *et al*., 2018a; Lin *et al*., 2019; Ostria‐Gallardo *et al*., 2020; Pardo *et al*., 2020; Montes *et al*., 2022). Under moderate water loss, expression patterns resemble typical drought responses, but as tissues approach a critical water potential, a distinct desiccation‐specific program is activated. The cue for this transition remains unclear but may involve hydration‐dependent biophysical signals, such as the phase separation of seed‐specific proteins like FLOE1 or SAM8 (Dorone *et al*., 2021; Wang *et al*., 2026).

Many of the most abundant transcripts in desiccating angiosperm tissues encode classical seed dehydration proteins, including LEAs, HSPs, ELIPs, oleosins, and enzymes involved in sugar metabolism (Cushman & Oliver, 2011; VanBuren *et al*., 2017; Costa *et al*., 2017b; Pardo *et al*., 2020). This overlap suggests that vegetative desiccation tolerance in angiosperms likely evolved through the reactivation or expansion of pre‐existing seed pathways, rather than through the *de novo* evolution of novel stress mechanisms (Oliver *et al*., 2000; Alpert, 2005; Gaff & Oliver, 2013; Marks *et al*., 2021a). Nearly all angiosperms produce desiccation‐tolerant reproductive structures, such as seeds and pollen, and comparative studies confirm that many protective genes and processes are shared between desiccation‐tolerant leaves and desiccation‐tolerant seeds. In resurrection grasses, desiccation‐induced genes are enriched for ABI5 binding motifs, linking stress responses in leaves to canonical seed maturation networks. Additionally, a novel drought‐responsive *cis*‐element upstream of ELIPs in *Oropetium thomaeum* suggests regulatory neofunctionalization, enabling activation of photoprotective genes under desiccation stress (St Aubin *et al*., 2022). In *Xerophyta humilis* and other resurrection plants, the canonical seed maturation regulators (e.g. *LEC1*, *ABI3*, *FUS3*, and *LEC2*) are not upregulated in vegetative tissues during drying, even though their downstream targets are strongly induced (Lyall *et al*., 2020). This points to a model where alternative regulators have been recruited to drive a seed‐like transcriptional program in leaves to rewire latent, ancestral mechanisms that were never entirely lost.

Many seed‐associated desiccation pathways are also activated in desiccation‐sensitive species under severe or lethal drought stress, but often as part of a broad, poorly coordinated stress response (Pardo *et al*., 2020). In resurrection plants, these same or similar pathways are deployed with greater coordination, timing, and magnitude, allowing vegetative tissues to prepare for drying and recover after rehydration. Thus, the repeated emergence of vegetative desiccation tolerance may depend less on novel protective mechanisms than on regulatory rewiring that reactivates and coordinates existing stress‐response pathways in the appropriate tissues and developmental context.

In desiccation‐tolerant organisms, a component of this coordination is the downregulation of metabolic processes, which limits the accumulation of ROS and other toxic byproducts. Evidence from diverse lineages suggests that desiccation tolerance involves a coordinated transition to a low‐metabolic or quiescent state. For example, desiccation‐tolerant green algae are distinguished from their desiccation‐sensitive relatives by the downregulation of photosynthesis, starch metabolism, and generation of metabolic intermediates (Peredo & Cardon, 2020). Similarly, the shutdown of the circadian clock during desiccation in the resurrection grass *Eragrostis nindensis* may indicate that broader regulatory networks are actively reprogrammed to facilitate entry into a quiescent state (Pardo, 2022). Vegetative desiccation tolerance may have re‐evolved by re‐coordinating latent programs that enable a rapid and effective transition into this protected state under severe water deficit.

The extent to which this regulatory control is dynamic or constitutive varies across lineages. Many desiccation‐tolerant bryophytes maintain high basal expression of protective genes and antioxidants even when hydrated, minimizing the need for transcriptional reprogramming during drying. Because these poikilohydric species can desiccate rapidly in response to abrupt changes in humidity or water availability, they often persist in a continuously primed state for desiccation and rehydration (Proctor *et al*., 2007; Alejo‐Jacuinde *et al*., 2025). By contrast, angiosperms typically experience more gradual declines in water availability, allowing time for inducible gene expression programs that are activated at low water potentials (Pardo *et al*., 2020). Differences among lineages reflect variation in how these programs are deployed, on a spectrum from constitutive preparedness in poikilohydric species to tightly regulated, inducible responses in angiosperms. In both cases, desiccation tolerance emerges from coordinated control of existing molecular machinery, reinforcing the view that regulatory architecture, rather than gene content alone, is the primary driver for the evolution of this complex trait.

## IV. Biotechnological applications of desiccation tolerance to crops and beyond

The extraordinary adaptations of desiccation‐tolerant organisms offer a rich reservoir of insights into how life persists under extreme abiotic stress. These survival strategies illustrate the outer limits of plant resilience but come with significant trade‐offs. Resurrection plants often exhibit reduced metabolic rates, slower biomass accumulation, and lower competitive ability, which limits the feasibility of engineering crops that are fully desiccation‐tolerant (Farrant & Hilhorst, 2022). Consequently, the challenge is not to recreate desiccation tolerance in a sensitive crop, but to translate this knowledge into practical applications. What can we learn from resurrection plants, and how can we use that information to enhance the resilience of crops without compromising yield or fitness? Rather than replicating desiccation tolerance, a more promising approach is to introduce or enhance key protective mechanisms, such as stress‐responsive transcription factors, osmoprotective sugars, antioxidants, and structural proteins into crop plants to improve their stress tolerance. Beyond agriculture, these dry‐state survival strategies may also inform applications in biotechnology and medicine, including improved preservation of biological materials.

### 1. Mechanistic approaches to crop improvement

Despite limitations on engineering a truly desiccation‐tolerant crop, many of the desiccation tolerance mechanisms discussed in this review can be leveraged for improved stress tolerance in sensitive organisms. Structural protectants show strong potential for crop improvement. Unlike regulatory elements, such as transcription factors, which can trigger wide‐ranging and sometimes deleterious shifts in gene expression, structural proteins like LEAs and sHSPs function primarily at the level of damage prevention and cellular stabilization and may cause fewer negative downstream effects. Photosynthetic protection also offers promising targets for improved tolerance in crops. Several pathways or proteins have already been applied in model and crop systems. Overexpressing trehalose synthesis genes or DT‐IDPs, such as sHSPs and LEAs, has enhanced water stress tolerance in *Arabidopsis*, tomato, rice, and tobacco (Garg *et al*., 2002; Cortina & Culiáñez‐Macià, 2005; Wang *et al*., 2006; Karim *et al*., 2007; Xiao *et al*., 2007; Yu *et al*., 2016). Additionally, elevating the expression of ELIPs increases abiotic stress tolerance (Zhuo *et al*., 2013). Upregulating alternative sugar biosynthetic pathways more commonly used in resurrection plants, such as sucrose or raffinose synthesis pathways, or minimizing photooxidative stress by enhancing regulation of Chl degradation and reassembly during stress may provide similar avenues for improving tolerance. Critically, many desiccation tolerance mechanisms are associated with inherent fitness costs, including growth penalties, altered carbon allocation, and reduced photosynthetic efficiency under nonstress conditions. These trade‐offs likely reflect the energetic and regulatory burden of maintaining a protective dry‐state ready physiology and help explain why desiccation tolerance has evolved in specialized ecological niches. Recognizing and managing these trade‐offs will be essential for translating desiccation tolerance mechanisms into crops, emphasizing strategies that deploy inducible or tissue‐specific protection rather than constitutive activation.

While modifying complex regulatory networks often risks unintended downstream consequences, many desiccation tolerance mechanisms are evolutionarily ancient, suggesting most plants may already harbor the genetic capacity for enhanced tolerance. Therefore, rather than introducing new genes, a more promising strategy could involve strategic reconfiguration of these pre‐existing pathways, such as abscisic acid, autophagy, or senescence. This may reduce the downstream effects associated with the introduction of foreign regulatory elements. For example, the ABA pathway is a central hub for drought and desiccation responses in vegetative tissues and seeds. The ABA receptor‐ligand interaction is well characterized (Nishimura *et al*., 2009), and synthetic ligands that mimic ABA could be applied as foliar sprays or soil additives, enabling the activation of ABA pathways without genetic engineering (Vaidya *et al*., 2019).

### 2. Applications of desiccation tolerance beyond agriculture

While much attention has focused on the applications of desiccation tolerance research for crop resilience, resurrection plants offer significant potential in pharmacology and biotechnology. Many species produce specialized metabolites with documented antioxidant, antiviral, and antibacterial activities (Gescher *et al*., 2011; Kamng'ona *et al*., 2011; Gechev *et al*., 2014; Bentley *et al*., 2019a,b; Djilianov *et al*., 2024). Although these properties are not necessarily unique to metabolites from resurrection plants, the same protective systems that preserve cellular integrity during prolonged drying can also contribute to the stability of these metabolites in the dry state. As a result, resurrection plants are a natural reservoir of biologically active compounds (Gescher *et al*., 2011; Kamng'ona *et al*., 2011; Gechev *et al*., 2014; Bentley *et al*., 2019a,b; Djilianov *et al*., 2024). For example, species like *Myrothamnus flabellifolia* and *Haberlea rhodopensis* produce polyphenols and glycosides with documented antioxidant, antiviral, and antibacterial properties, offering resources for the cosmetic and pharmaceutical industries (Gescher *et al*., 2011; Kamng'ona *et al*., 2011; Gechev *et al*., 2014; Bentley *et al*., 2019a,b; Djilianov *et al*., 2024). Given the vast and largely unexplored diversity of plant secondary metabolites, resurrection plants may represent an untapped source of novel bioactive compounds.

Furthermore, desiccation tolerance mechanisms hold promise for storage and transport of biological materials. Current gold‐standard storage methods for cells, tissues, vaccines, and medicines require flash freezing and ultralow temperatures, which are costly and logistically demanding, making distribution inconvenient and limiting the range of distribution. However, by leveraging the vitrification properties of trehalose and LEA proteins, which stabilize cellular structures without requiring low temperatures, researchers have successfully preserved mammalian cells, sperm, and plant tissues in a dehydrated state (Guo *et al*., 2000; Chen *et al*., 2001; Gordon *et al*., 2001; McGinnis *et al*., 2005; Czernik *et al*., 2020; Zamecnik *et al*., 2021). Transitioning to dry‐state storage could substantially improve the accessibility and distribution of medical supplies by eliminating the need for ultralow‐temperature infrastructure.

## V. Concluding thoughts

The growing wealth of literature suggests that organisms across the tree of life deploy similar molecular strategies to survive desiccation. However, research on desiccation tolerance has often been siloed within specific study systems, with limited cross‐disciplinary integration (Lotreck *et al*., 2024). As a result, conceptual advances in one lineage are not always translated into broader mechanistic frameworks, constraining synthesis across biological scales and kingdoms. Here, we aimed to integrate discoveries from desiccation‐tolerant plants with insights from other organisms, highlighting common mechanisms that appear to be broadly conserved across diverse lineages. These shared strategies have been repeatedly reactivated or repurposed during evolutionary transitions from aquatic to terrestrial environments, enabling survival in extreme habitats and the dormancy and dispersal of propagules, such as seeds, spores, and pollen. As comparative datasets continue to expand, it is becoming clear that desiccation tolerance is not governed by a single pathway but instead represents an emergent phenotype arising from the coordinated interaction between numerous cellular, physiological, and regulatory systems.

Despite more than 6000 published studies on desiccation tolerance, critical knowledge gaps remain. A major unresolved question is when desiccation tolerance evolved and whether the underlying mechanisms reflect ancient conserved pathways that were retained from early terrestrial ancestors or convergent solutions in which different genes and proteins provide similar protective functions. Distinguishing between these possibilities will require broader phylogenetic sampling and comparative analyses that integrate genomic, biochemical, and physiological datasets across kingdoms. In plants, we lack a complete understanding of the upstream regulatory signals that distinguish drought responses from desiccation tolerance. These signals may involve transcriptional or translational regulation, hormone signaling networks, or poorly understood biophysical cues associated with declining cellular hydration. The spatial and temporal coordination of these responses across tissues and cell types also remains largely unexplored. In addition, the extent of light‐induced damage and the role of photoprotective mechanisms in safeguarding macromolecules remain poorly characterized. More broadly, it remains unclear how the numerous protective pathways described here are integrated into regulatory networks that enable survival at extremely low water contents, and whether these networks represent a shared core architecture across desiccation‐tolerant organisms or lineage‐specific solutions to a common physiological challenge.

Expanding molecular and genomic toolkits offers promising avenues for exploring these open questions. Several resurrection plants have established transformation protocols (Furini *et al*., 1994; Tóth *et al*., 2006; Chowdhury *et al*., 2020), but mechanistic studies remain limited and CRISPR approaches are largely unexplored. Developing gene editing tools in these species would enable direct functional testing of candidate regulators and gene functions underlying desiccation tolerance. Extending these approaches to desiccation‐tolerant grasses could also facilitate translational applications in crop species. The increasing accessibility of single cell RNA sequencing further provides opportunities to resolve how distinct cell types coordinate desiccation responses across tissues. Finally, broader comparative evolutionary and systems biology analyses that include closely related species will help reconstruct the sequence in which key traits evolved and deepen our understanding of how desiccation tolerance emerged.

The exceptional resilience of desiccation‐tolerant organisms offers a framework for understanding survival at environmental extremes and a rich source of molecular strategies that have diverse practical applications. Improving plant resilience has been a longstanding challenge, as discoveries in the laboratory often fail to translate into improved performance in replicated field trials. Rather than engineering fully desiccation‐tolerant crops, biotechnological efforts can focus on targeting downstream protectants and endpoint metabolites. These molecules, shaped by evolution to function as cellular stabilizers, are attractive because they operate largely outside of complex regulatory cascades. Beyond agriculture, the principles of desiccation tolerance are now being applied to stabilize biologics, enable dry‐state storage of cells and vaccines, and inspire synthetic biology innovations. In this way, resurrection plants and other desiccation‐tolerant lineages serve as both evolutionary case studies and engineering guides.

## Competing interests

None declared.

## Author contributions

JS and RV conceived the review, prepared the figures, and wrote and edited the manuscript. Both authors approved the final version.

## Disclaimer

The New Phytologist Foundation remains neutral with regard to jurisdictional claims in maps and in any institutional affiliations.

## References

[nph71466-bib-0001] Adams RP , Kendall E , Kartha KK . 1990. Comparison of free sugars in growing and desiccated plants of *Selaginella lepidophylla* . Biochemical Systematics and Ecology 18: 107–110.

[nph71466-bib-0002] Adhikari BN , Wall DH , Adams BJ . 2009. Desiccation survival in an Antarctic nematode: molecular analysis using expressed sequenced tags. BMC Genomics 10: 69. 19203352 10.1186/1471-2164-10-69 PMC2667540

[nph71466-bib-0003] Aghaie P , Tafreshi SAH . 2020. Central role of 70‐kDa heat shock protein in adaptation of plants to drought stress. Cell Stress & Chaperones 25: 1071–1081. 32720054 10.1007/s12192-020-01144-7 PMC7591640

[nph71466-bib-0004] Alamillo J , Almoguera C , Bartels D , Jordano J . 1995. Constitutive expression of small heat shock proteins in vegetative tissues of the resurrection plant *Craterostigma plantagineum* . Plant Molecular Biology 29: 1093–1099. 8555452 10.1007/BF00014981

[nph71466-bib-0005] Alejo‐Jacuinde G , Chávez Montes RA , Gutierrez Reyes CD , Yong‐Villalobos L , Simpson J , Herrera‐Estrella L . 2025. Gene family rearrangements and transcriptional priming drive the evolution of vegetative desiccation tolerance in *Selaginella* . The Plant Journal 121: e17169. 39666518 10.1111/tpj.17169 PMC11711927

[nph71466-bib-0006] Alpert P . 2005. The limits and frontiers of desiccation‐tolerant life. Integrative and Comparative Biology 45: 685–695. 21676818 10.1093/icb/45.5.685

[nph71466-bib-0007] Alpert P . 2006. Constraints of tolerance: why are desiccation‐tolerant organisms so small or rare? The Journal of Experimental Biology 209: 1575–1584. 16621938 10.1242/jeb.02179

[nph71466-bib-0008] Azar M , Goldbecker E , Karpovsky D , Shpilman M , Breker M , de Vries J , Mosquna A . 2025. Shared abscisic acid biosynthesis pathway across 600 million years of streptophyte evolution. Plant Physiology 198: kiaf121. 40415190 10.1093/plphys/kiaf121

[nph71466-bib-0009] Banerjee A , Roychoudhury A . 2016. Group II late embryogenesis abundant (LEA) proteins: structural and functional aspects in plant abiotic stress. Plant Growth Regulation 79: 1–17.

[nph71466-bib-0010] Barták M , Solhaug KA , Vráblíková H , Gauslaa Y . 2006. Curling during desiccation protects the foliose lichen *Lobaria pulmonaria* against photoinhibition. Oecologia 149: 553–560. 16804701 10.1007/s00442-006-0476-2

[nph71466-bib-0011] Bartels D , Giarola V , Chandler J . 2025. Unravelling the molecular network of desiccation tolerance in resurrection plants started with the model plant *Craterostigma plantagineum* . Planta 262: 37. 40560488 10.1007/s00425-025-04752-8 PMC12198318

[nph71466-bib-0012] Bartels D , Schneider K , Terstappen G , Piatkowski D , Salamini F . 1990. Molecular cloning of abscisic acid‐modulated genes which are induced during desiccation of the resurrection plant *Craterostigma plantagineum* . Planta 181: 27–34. 24196671 10.1007/BF00202321

[nph71466-bib-0013] Bellafiore S , Barneche F , Peltier G , Rochaix J‐D . 2005. State transitions and light adaptation require chloroplast thylakoid protein kinase STN7. Nature 433: 892–895. 15729347 10.1038/nature03286

[nph71466-bib-0014] Belott CJ , Gusev OA , Kikawada T , Menze MA . 2024. Membraneless and membrane‐bound organelles in an anhydrobiotic cell line are protected from desiccation‐induced damage. Cell Stress & Chaperones 29: 425–436. 38608858 10.1016/j.cstres.2024.04.002 PMC11061232

[nph71466-bib-0015] Bentley J , Moore JP , Farrant JM . 2019a. Metabolomics as a complement to phylogenetics for assessing intraspecific boundaries in the desiccation‐tolerant medicinal shrub *Myrothamnus flabellifolia* (Myrothamnaceae). Phytochemistry 159: 127–136. 30611872 10.1016/j.phytochem.2018.12.016

[nph71466-bib-0016] Bentley J , Moore JP , Farrant JM . 2019b. Metabolomic profiling of the desiccation‐tolerant medicinal shrub *Myrothamnus flabellifolia* indicates phenolic variability across its natural habitat: implications for tea and cosmetics production. Molecules 24: 1240. 30934961 10.3390/molecules24071240 PMC6479747

[nph71466-bib-0017] Besagni C , Kessler F . 2013. A mechanism implicating plastoglobules in thylakoid disassembly during senescence and nitrogen starvation. Planta 237: 463–470. 23187680 10.1007/s00425-012-1813-9

[nph71466-bib-0018] Bewley JD . 1979. Physiological aspects of desiccation tolerance. Annual Review of Plant Physiology 30: 195–238.

[nph71466-bib-0019] Bianchi G , Gamba A , Murelli C , Salamini F , Bartels D . 1991. Novel carbohydrate metabolism in the resurrection plant *Craterostigma plantagineum* . The Plant Journal 1: 355–359. 29345773 10.1046/j.1365-313X.1991.t01-11-00999.x

[nph71466-bib-0020] Boothby TC , Pielak GJ . 2017. Intrinsically disordered proteins and desiccation tolerance: elucidating functional and mechanistic underpinnings of anhydrobiosis. BioEssays 39: 1700119. 10.1002/bies.201700119 28901557

[nph71466-bib-0021] Boothby TC , Tapia H , Brozena AH , Piszkiewicz S , Smith AE , Giovannini I , Rebecchi L , Pielak GJ , Koshland D , Goldstein B . 2017. Tardigrades use intrinsically disordered proteins to survive desiccation. Molecular Cell 65: 975–984. 28306513 10.1016/j.molcel.2017.02.018 PMC5987194

[nph71466-bib-0022] Buitink J , Leprince O . 2004. Glass formation in plant anhydrobiotes: survival in the dry state. Cryobiology 48: 215–228. 15157771 10.1016/j.cryobiol.2004.02.011

[nph71466-bib-0023] Carey SB , Jenkins J , Lovell JT , Maumus F , Sreedasyam A , Payton AC , Shu S , Tiley GP , Fernandez‐Pozo N , Barry K *et al*. 2020. The Ceratodon purpureus genome uncovers structurally complex, gene rich sex chromosomes. *bioRxiv* . doi: 10.1101/2020.07.03.163634.

[nph71466-bib-0024] Charuvi D , Nevo R , Aviv‐Sharon E , Gal A , Kiss V , Shimoni E , Farrant JM , Kirchhoff H , Reich Z . 2019. Chloroplast breakdown during dehydration of a homoiochlorophyllous resurrection plant proceeds via senescence‐like processes. Environmental and Experimental Botany 157: 100–111.

[nph71466-bib-0025] Charuvi D , Nevo R , Shimoni E , Naveh L , Zia A , Adam Z , Farrant JM , Kirchhoff H , Reich Z . 2015. Photoprotection conferred by changes in photosynthetic protein levels and organization during dehydration of a homoiochlorophyllous resurrection plant. Plant Physiology 167: 1554–1565. 25713340 10.1104/pp.114.255794 PMC4378169

[nph71466-bib-0026] Chen H , Chu P , Zhou Y , Li Y , Liu J , Ding Y , Tsang EWT , Jiang L , Wu K , Huang S . 2012. Overexpression of AtOGG1, a DNA glycosylase/AP lyase, enhances seed longevity and abiotic stress tolerance in Arabidopsis. Journal of Experimental Botany 63: 4107–4121. 22473985 10.1093/jxb/ers093

[nph71466-bib-0027] Chen T , Acker JP , Eroglu A , Cheley S , Bayley H , Fowler A , Toner M . 2001. Beneficial effect of intracellular trehalose on the membrane integrity of dried mammalian cells. Cryobiology 43: 168–181. 11846471 10.1006/cryo.2001.2360

[nph71466-bib-0028] Chowdhury SR , Hoque S , Akter N . 2020. Optimization of regeneration and *Agrobacterium tumefaciens*‐mediated transient transformation systems for Australian native extremophile, *Tripogon loliiformis* . Journal of King Saud University, Science 32: 3476–3485.

[nph71466-bib-0029] Christ B , Egert A , Süssenbacher I , Kräutler B , Bartels D , Peters S , Hörtensteiner S . 2014. Water deficit induces chlorophyll degradation via the ‘PAO/phyllobilin’ pathway in leaves of homoio‐ (*Craterostigma pumilum*) and poikilochlorophyllous (*Xerophyta viscosa*) resurrection plants: chlorophyll breakdown in resurrection plants. Plant, Cell & Environment 37: 2521–2531. 10.1111/pce.12308 24697723

[nph71466-bib-0030] Clegg JS , Seitz P , Seitz W , Hazlewood CF . 1982. Cellular responses to extreme water loss: the water‐replacement hypothesis. Cryobiology 19: 306–316. 7105783 10.1016/0011-2240(82)90159-6

[nph71466-bib-0031] Cooper K , Farrant JM . 2002. Recovery of the resurrection plant *Craterostigma wilmsii* from desiccation: protection versus repair. Journal of Experimental Botany 53: 1805–1813. 12147731 10.1093/jxb/erf028

[nph71466-bib-0032] Cortina C , Culiáñez‐Macià FA . 2005. Tomato abiotic stress enhanced tolerance by trehalose biosynthesis. Plant Science 169: 75–82.

[nph71466-bib-0033] Costa M‐CD , Artur MAS , Maia J , Jonkheer E , Derks MFL , Nijveen H , Williams B , Mundree SG , Jiménez‐Gómez JM , Hesselink T *et al*. 2017a. A footprint of desiccation tolerance in the genome of *Xerophyta viscosa* . Nature Plants 3: 17038. 28346448 10.1038/nplants.2017.38

[nph71466-bib-0034] Costa M‐CD , Cooper K , Hilhorst HWM , Farrant JM . 2017b. Orthodox seeds and resurrection plants: two of a kind? Plant Physiology 175: 589–599. 28851758 10.1104/pp.17.00760 PMC5619911

[nph71466-bib-0035] Crowe JH . 1971. Anhydrobiosis: an unsolved problem. The American Naturalist 105: 563–573.

[nph71466-bib-0036] Crowe JH , Carpenter JF , Crowe LM . 1998a. The role of vitrification in anhydrobiosis. Annual Review of Physiology 60: 73–103. 10.1146/annurev.physiol.60.1.73 9558455

[nph71466-bib-0037] Crowe JH , Clegg JS , Crowe LM . 1998b. Anhydrobiosis: the water replacement hypothesis. In: The properties of water in foods ISOPOW 6. Boston, MA, USA: Springer US, 440–455.

[nph71466-bib-0038] Cushman JC , Oliver MJ . 2011. Understanding vegetative desiccation tolerance using integrated functional genomics approaches within a comparative evolutionary framework. In: Ecological studies: analysis and synthesis. Berlin, Heidelberg, New York NY. Plant desiccation tolerance. Berlin, Heidelberg, Germany: Springer Berlin Heidelberg, 307–338.

[nph71466-bib-0039] Czernik M , Fidanza A , Luongo FP , Valbonetti L , Scapolo PA , Patrizio P , Loi P . 2020. Late embryogenesis abundant (LEA) proteins confer water stress tolerance to mammalian somatic cells. Cryobiology 92: 189–196. 31952948 10.1016/j.cryobiol.2020.01.009

[nph71466-bib-0040] Dace H , Sherwin HW , Illing N , Farrant JM . 1998. Use of metabolic inhibitors to elucidate mechanisms of recovery from desiccation stress in the resurrection plant *Xerophyta humilis* . Plant Growth Regulation 24: 171–177.

[nph71466-bib-0041] Dace HJ , Adetunji AE , Moore JP , Farrant JM , Hilhorst HW . 2023. A review of the role of metabolites in vegetative desiccation tolerance of angiosperms. Current Opinion in Plant Biology 75: 102410. 37413962 10.1016/j.pbi.2023.102410

[nph71466-bib-0042] Dadras A , Duminil P , de Vries S , Irisarri I , Feussner I , de Vries J . 2025. Algal origins of core land plant stress response subnetworks. The Plant Journal 122: e70291. 40550109 10.1111/tpj.70291 PMC12185132

[nph71466-bib-0043] Del Casino C , Conti V , Licata S , Cai G , Cantore A , Ricci C , Cantara S . 2024. Mitigation of UV‐B radiation stress in tobacco pollen by expression of the tardigrade damage suppressor protein (Dsup). Cells 13: 840. 38786062 10.3390/cells13100840 PMC11119994

[nph71466-bib-0044] Dinakar C , Bartels D . 2012. Light response, oxidative stress management and nucleic acid stability in closely related Linderniaceae species differing in desiccation tolerance. Planta 236: 541–555. 22437647 10.1007/s00425-012-1628-8

[nph71466-bib-0045] Djilianov D , Moyankova D , Mladenov P , Topouzova‐Hristova T , Kostadinova A , Staneva G , Zasheva D , Berkov S , Simova‐Stoilova L . 2024. Resurrection plants‐A valuable source of natural bioactive compounds: from word‐of‐mouth to scientifically proven sustainable use. Metabolites 14: 113. 38393005 10.3390/metabo14020113 PMC10890500

[nph71466-bib-0046] Dorone Y , Boeynaems S , Flores E , Jin B , Hateley S , Bossi F , Lazarus E , Pennington JG , Michiels E , De Decker M *et al*. 2021. A prion‐like protein regulator of seed germination undergoes hydration‐dependent phase separation. Cell 184: 4284–4298. 34233164 10.1016/j.cell.2021.06.009 PMC8513799

[nph71466-bib-0047] Egert A , Eicher B , Keller F , Peters S . 2015. Evidence for water deficit‐induced mass increases of raffinose family oligosaccharides (RFOs) in the leaves of three Craterostigma resurrection plant species. Frontiers in Physiology 6: 206. 26257658 10.3389/fphys.2015.00206 PMC4510996

[nph71466-bib-0048] Elliott B , Haltiwanger RS , Futcher B . 1996. Synergy between trehalose and Hsp104 for thermotolerance in *Saccharomyces cerevisiae* . Genetics 144: 923–933. 8913738 10.1093/genetics/144.3.923 PMC1207632

[nph71466-bib-0049] Farrant JM . 2000. A comparison of mechanisms of desiccation tolerance among three angiosperm resurrection plant species. Plant Ecology 151: 29–39.

[nph71466-bib-0050] Farrant JM , Hilhorst H . 2022. Crops for dry environments. Current Opinion in Biotechnology 74: 84–91. 34808476 10.1016/j.copbio.2021.10.026

[nph71466-bib-0051] Farrant JM , Vander Willigen C , Loffell DA , Bartsch S , Whittaker A . 2003. An investigation into the role of light during desiccation of three angiosperm resurrection plants. Plant, Cell & Environment 26: 1275–1286.

[nph71466-bib-0052] Feng X , Zheng J , Irisarri I , Yu H , Zheng B , Ali Z , de Vries S , Keller J , Fürst‐Jansen JMR , Dadras A *et al*. 2024. Genomes of multicellular algal sisters to land plants illuminate signaling network evolution. Nature Genetics 56: 1018–1031. 38693345 10.1038/s41588-024-01737-3 PMC11096116

[nph71466-bib-0053] Fleming ED , Castenholz RW . 2007. Effects of periodic desiccation on the synthesis of the UV‐screening compound, scytonemin, in cyanobacteria. Environmental Microbiology 9: 1448–1455. 17504482 10.1111/j.1462-2920.2007.01261.x

[nph71466-bib-0054] Furini A , Koncz C , Salamini F , Bartels D . 1994. Agrobacterium‐mediated transformation of the desiccation‐tolerant plant *Craterostigma plantagineum* . Plant Cell Reports 14: 102–106. 24192874 10.1007/BF00233770

[nph71466-bib-0055] Gaff DF . 1977. Desiccation tolerant vascular plants of southern Africa. Oecologia 31: 95–109. 28309154 10.1007/BF00348713

[nph71466-bib-0056] Gaff DF . 1987. Desiccation tolerant plants in South America. Oecologia 74: 133–136. 28310426 10.1007/BF00377357

[nph71466-bib-0057] Gaff DF , Bole PV . 1986. Resurrection grasses in India. Oecologia 71: 159–160. 28312100 10.1007/BF00377337

[nph71466-bib-0058] Gaff DF , Oliver M . 2013. The evolution of desiccation tolerance in angiosperm plants: a rare yet common phenomenon. Functional Plant Biology 40: 315–328. 32481110 10.1071/FP12321

[nph71466-bib-0059] Gao B , Li X , Liang Y , Chen M , Liu H , Liu Y , Wang J , Zhang J , Zhang Y , Oliver MJ *et al*. 2024. Drying without dying: a genome database for desiccation‐tolerant plants and evolution of desiccation tolerance. Plant Physiology 194: 2249–2262. 38109500 10.1093/plphys/kiad672

[nph71466-bib-0060] Gao Z‐Y , Li Z‐H , Lin D‐L , Jin X‐H . 2021. Chromosome‐scale genome assembly of the resurrection plant *Acanthochlamys bracteata* (Velloziaceae). Genome Biology and Evolution 13: evab147. 34165527 10.1093/gbe/evab147 PMC8358219

[nph71466-bib-0061] Garg AK , Kim J‐K , Owens TG , Ranwala AP , Choi YD , Kochian LV , Wu RJ . 2002. Trehalose accumulation in rice plants confers high tolerance levels to different abiotic stresses. Proceedings of the National Academy of Sciences, USA 99: 15898–15903. 10.1073/pnas.252637799 PMC138536 12456878

[nph71466-bib-0062] Gauslaa Y , Coxson DS , Solhaug KA . 2012. The paradox of higher light tolerance during desiccation in rare old forest cyanolichens than in more widespread co‐occurring chloro‐ and cephalolichens. New Phytologist 195: 812–822. 22762452 10.1111/j.1469-8137.2012.04221.x PMC3593164

[nph71466-bib-0063] Gechev T , Lyall R , Petrov V , Bartels D . 2021. Systems biology of resurrection plants. Cellular and Molecular Life Sciences 78: 6365–6394. 34390381 10.1007/s00018-021-03913-8 PMC8558194

[nph71466-bib-0064] Gechev TS , Hille J , Woerdenbag HJ , Benina M , Mehterov N , Toneva V , Fernie AR , Mueller‐Roeber B . 2014. Natural products from resurrection plants: potential for medical applications. Biotechnology Advances 32: 1091–1101. 24681091 10.1016/j.biotechadv.2014.03.005

[nph71466-bib-0065] Georgieva K , Dagnon S , Gesheva E , Bojilov D , Mihailova G , Doncheva S . 2017. Antioxidant defense during desiccation of the resurrection plant *Haberlea rhodopensis* . Plant Physiology and Biochemistry 114: 51–59. 28268193 10.1016/j.plaphy.2017.02.021

[nph71466-bib-0066] Georgieva K , Röding A , Büchel C . 2009. Changes in some thylakoid membrane proteins and pigments upon desiccation of the resurrection plant *Haberlea rhodopensis* . Journal of Plant Physiology 166: 1520–1528. 19428140 10.1016/j.jplph.2009.03.010

[nph71466-bib-0067] Gescher K , Kühn J , Lorentzen E , Hafezi W , Derksen A , Deters A , Hensel A . 2011. Proanthocyanidin‐enriched extract from *Myrothamnus flabellifolia* Welw. exerts antiviral activity against herpes simplex virus type 1 by inhibition of viral adsorption and penetration. Journal of Ethnopharmacology 134: 468–474. 21211557 10.1016/j.jep.2010.12.038

[nph71466-bib-0068] Giovannini I , Corsetto PA , Altiero T , Montorfano G , Guidetti R , Rizzo AM , Rebecchi L . 2022. Antioxidant response during the kinetics of anhydrobiosis in two eutardigrade species. Life 12: 817. 35743848 10.3390/life12060817 PMC9225123

[nph71466-bib-0069] Gladyshev E , Meselson M . 2008. Extreme resistance of bdelloid rotifers to ionizing radiation. Proceedings of the National Academy of Sciences, USA 105: 5139–5144. 10.1073/pnas.0800966105 PMC2278216 18362355

[nph71466-bib-0070] Gladyshev EA , Arkhipova IR . 2010. Genome structure of bdelloid rotifers: shaped by asexuality or desiccation? The Journal of Heredity 101: S85–S93. 20421328 10.1093/jhered/esq008

[nph71466-bib-0071] Gordon SL , Oppenheimer SR , Mackay AM , Brunnabend J , Puhlev I , Levine F . 2001. Recovery of human mesenchymal stem cells following dehydration and rehydration. Cryobiology 43: 182–187. 11846472 10.1006/cryo.2001.2361

[nph71466-bib-0072] Guo N , Puhlev I , Brown DR , Mansbridge J , Levine F . 2000. Trehalose expression confers desiccation tolerance on human cells. Nature Biotechnology 18: 168–171. 10.1038/72616 10657122

[nph71466-bib-0073] Gupta S , Petrov V , Garg V , Mueller‐Roeber B , Fernie AR , Nikoloski Z , Gechev T . 2024. The genome of *Haberlea rhodopensis* provides insights into the mechanisms for tolerance to multiple extreme environments. Cellular and Molecular Life Sciences 81: 117. 38443747 10.1007/s00018-024-05140-3 PMC10914886

[nph71466-bib-0074] Gusev O , Nakahara Y , Vanyagina V , Malutina L , Cornette R , Sakashita T , Hamada N , Kikawada T , Kobayashi Y , Okuda T . 2010. Anhydrobiosis‐associated nuclear DNA damage and repair in the sleeping chironomid: linkage with radioresistance. PLoS ONE 5: e14008. 21103355 10.1371/journal.pone.0014008 PMC2982815

[nph71466-bib-0075] Gusev O , Suetsugu Y , Cornette R , Kawashima T , Logacheva MD , Kondrashov AS , Penin AA , Hatanaka R , Kikuta S , Shimura S *et al*. 2014. Comparative genome sequencing reveals genomic signature of extreme desiccation tolerance in the anhydrobiotic midge. Nature Communications 5: 4784. 10.1038/ncomms5784 PMC4175575 25216354

[nph71466-bib-0076] Hashimoto T , Horikawa DD , Saito Y , Kuwahara H , Kozuka‐Hata H , Shin‐I T , Minakuchi Y , Ohishi K , Motoyama A , Aizu T *et al*. 2016. Extremotolerant tardigrade genome and improved radiotolerance of human cultured cells by tardigrade‐unique protein. Nature Communications 7: 12808. 10.1038/ncomms12808 PMC5034306 27649274

[nph71466-bib-0077] Haslbeck M , Vierling E . 2015. A first line of stress defense: small heat shock proteins and their function in protein homeostasis. Journal of Molecular Biology 427: 1537–1548. 25681016 10.1016/j.jmb.2015.02.002 PMC4360138

[nph71466-bib-0078] Hibshman JD , Carra S , Goldstein B . 2023. Tardigrade small heat shock proteins can limit desiccation‐induced protein aggregation. Communications Biology 6: 121. 36717706 10.1038/s42003-023-04512-y PMC9887055

[nph71466-bib-0079] Hibshman JD , Clegg JS , Goldstein B . 2020. Mechanisms of desiccation tolerance: themes and variations in brine shrimp, roundworms, and tardigrades. Frontiers in Physiology 11: 592016. 33192606 10.3389/fphys.2020.592016 PMC7649794

[nph71466-bib-0080] Hincha DK , Zuther E , Heyer AG . 2003. The preservation of liposomes by raffinose family oligosaccharides during drying is mediated by effects on fusion and lipid phase transitions. Biochimica et Biophysica Acta 1612: 172–177. 12787935 10.1016/s0005-2736(03)00116-0

[nph71466-bib-0081] Hoekstra FA , Golovina EA , Buitink J . 2001. Mechanisms of plant desiccation tolerance. Trends in Plant Science 6: 431–438. 11544133 10.1016/s1360-1385(01)02052-0

[nph71466-bib-0082] Hutin C , Nussaume L , Moise N , Moya I , Kloppstech K , Havaux M . 2003. Early light‐induced proteins protect Arabidopsis from photooxidative stress. Proceedings of the National Academy of Sciences, USA 100: 4921–4926. 10.1073/pnas.0736939100 PMC153656 12676998

[nph71466-bib-0083] Ingle RA , Collett H , Cooper K , Takahashi Y , Farrant JM , Illing N . 2008. Chloroplast biogenesis during rehydration of the resurrection plant *Xerophyta humilis*: parallels to the etioplast‐chloroplast transition. Plant, Cell & Environment 31: 1813–1824. 10.1111/j.1365-3040.2008.01887.x 18771571

[nph71466-bib-0084] Ingle RA , Schmidt UG , Farrant JM , Thomson JA , Mundree SG . 2007. Proteomic analysis of leaf proteins during dehydration of the resurrection plant *Xerophyta viscosa* . Plant, Cell & Environment 30: 435–446. 10.1111/j.1365-3040.2006.01631.x 17324230

[nph71466-bib-0085] Jiang G , Wang Z , Shang H , Yang W , Hu Z , Phillips J , Deng X . 2007. Proteome analysis of leaves from the resurrection plant *Boea hygrometrica* in response to dehydration and rehydration. Planta 225: 1405–1420. 17160389 10.1007/s00425-006-0449-z

[nph71466-bib-0086] Kamng'ona A , Moore JP , Lindsey G , Brandt W . 2011. Inhibition of HIV‐1 and M‐MLV reverse transcriptases by a major polyphenol (3,4,5 tri‐O‐galloylquinic acid) present in the leaves of the South African resurrection plant, *Myrothamnus flabellifolia* . Journal of Enzyme Inhibition and Medicinal Chemistry 26: 843–853. 21443452 10.3109/14756366.2011.566220

[nph71466-bib-0087] Karim S , Aronsson H , Ericson H , Pirhonen M , Leyman B , Welin B , Mäntylä E , Palva ET , Van Dijck P , Holmström K‐O . 2007. Improved drought tolerance without undesired side effects in transgenic plants producing trehalose. Plant Molecular Biology 64: 371–386. 17453154 10.1007/s11103-007-9159-6

[nph71466-bib-0088] Kim SX , Çamdere G , Hu X , Koshland D , Tapia H . 2018. Synergy between the small intrinsically disordered protein Hsp12 and trehalose sustain viability after severe desiccation. eLife 7: e38337. 30010539 10.7554/eLife.38337 PMC6054528

[nph71466-bib-0089] Kirke J , Jin X‐L , Zhang X‐H . 2020. Expression of a tardigrade Dsup gene enhances genome protection in plants. Molecular Biotechnology 62: 563–571. 32955680 10.1007/s12033-020-00273-9

[nph71466-bib-0090] Koster KL . 1991. Glass formation and desiccation tolerance in seeds. Plant Physiology 96: 302–304. 16668169 10.1104/pp.96.1.302 PMC1080750

[nph71466-bib-0091] Kranner I , Beckett R , Hochman A , Nash TH III . 2008. Desiccation‐tolerance in lichens: a review. The Bryologist 111: 576–593.

[nph71466-bib-0092] Kranner I , Beckett RP , Wornik S , Zorn M , Pfeifhofer HW . 2002. Revival of a resurrection plant correlates with its antioxidant status: antioxidants during revival of a resurrection plant. The Plant Journal 31: 13–24. 12100479 10.1046/j.1365-313x.2002.01329.x

[nph71466-bib-0093] Kranner I , Birtic S . 2005. A modulating role for antioxidants in desiccation tolerance. Integrative and Comparative Biology 45: 734–740. 21676824 10.1093/icb/45.5.734

[nph71466-bib-0094] Leprince O , Buitink J . 2015. Introduction to desiccation biology: from old borders to new frontiers. Planta 242: 369–378. 26142353 10.1007/s00425-015-2357-6

[nph71466-bib-0095] Li J , Harper LC , Golubovskaya I , Wang CR , Weber D , Meeley RB , McElver J , Bowen B , Cande WZ , Schnable PS . 2007. Functional analysis of maize RAD51 in meiosis and double‐strand break repair. Genetics 176: 1469–1482. 17507687 10.1534/genetics.106.062604 PMC1931559

[nph71466-bib-0096] Lin C , Jia S‐N , Yang F , Jia W‐H , Yu X‐J , Yang J‐S , Yang W‐J . 2016. The transcription factor p8 regulates autophagy during diapause embryo formation in *Artemia parthenogenetica* . Cell Stress & Chaperones 21: 665–675. 27125785 10.1007/s12192-016-0692-6 PMC4907998

[nph71466-bib-0097] Lin C‐T , Xu T , Xing S‐L , Zhao L , Sun R‐Z , Liu Y , Moore JP , Deng X . 2019. Weighted gene co‐expression network analysis (WGCNA) reveals the hub role of protein ubiquitination in the acquisition of desiccation tolerance in *Boea hygrometrica* . Plant & Cell Physiology 60: 2707–2719. 31410481 10.1093/pcp/pcz160

[nph71466-bib-0098] Liu J , Liu J , Deng L , Liu H , Liu H , Zhao W , Zhao Y , Sun X , Fan S , Wang H *et al*. 2023. An intrinsically disordered region‐containing protein mitigates the drought‐growth trade‐off to boost yields. Plant Physiology 192: 274–292. 36746783 10.1093/plphys/kiad074 PMC10152686

[nph71466-bib-0099] Liu S , Fang S , Cong B , Li T , Yi D , Zhang Z , Zhao L , Zhang P . 2022. The Antarctic moss *Pohlia nutans* genome provides insights into the evolution of bryophytes and the adaptation to extreme terrestrial habitats. Frontiers in Plant Science 13: 920138. 35783932 10.3389/fpls.2022.920138 PMC9247546

[nph71466-bib-0100] Lohaus R , Van de Peer Y . 2016. Of dups and dinos: evolution at the K/Pg boundary. Current Opinion in Plant Biology 30: 62–69. 26894611 10.1016/j.pbi.2016.01.006

[nph71466-bib-0101] Lotreck SG , Ghassemi M , VanBuren RT . 2024. Unifying the research landscape of desiccation tolerance to identify trends, gaps, and opportunities. *bioRxiv* . doi: 10.1101/2024.06.06.597802.

[nph71466-bib-0102] Lyall R , Schlebusch SA , Proctor J , Prag M , Hussey SG , Ingle RA , Illing N . 2020. Vegetative desiccation tolerance in the resurrection plant *Xerophyta humilis* has not evolved through reactivation of the seed canonical LAFL regulatory network. The Plant Journal 101: 1349–1367. 31680354 10.1111/tpj.14596 PMC7187197

[nph71466-bib-0103] Macovei A , Balestrazzi A , Confalonieri M , Faé M , Carbonera D . 2011. New insights on the barrel medic MtOGG1 and MtFPG functions in relation to oxidative stress response *in planta* and during seed imbibition. Plant Physiology and Biochemistry 49: 1040–1050. 21696973 10.1016/j.plaphy.2011.05.007

[nph71466-bib-0104] MacRae TH . 2016. Stress tolerance during diapause and quiescence of the brine shrimp, Artemia. Cell Stress & Chaperones 21: 9–18. 26334984 10.1007/s12192-015-0635-7 PMC4679736

[nph71466-bib-0105] Marcu D , Damian G , Cosma C , Cristea V . 2013. Gamma radiation effects on seed germination, growth and pigment content, and ESR study of induced free radicals in maize (*Zea mays*). Journal of Biological Physics 39: 625–634. 23996407 10.1007/s10867-013-9322-z PMC3758825

[nph71466-bib-0106] Marks RA , Delgado P , Makonya GM , Cooper K , VanBuren R , Farrant JM . 2024a. Higher order polyploids exhibit enhanced desiccation tolerance in the grass *Microchloa caffra* . Journal of Experimental Botany 75: 3612–3623. 38511472 10.1093/jxb/erae126 PMC11156804

[nph71466-bib-0107] Marks RA , Ekwealor JTB , Artur MAS , Bondi L , Boothby TC , Carmo OMS , Centeno DC , Coe KK , Dace HJW , Field S *et al*. 2025a. Life on the dry side: a roadmap to understanding desiccation tolerance and accelerating translational applications. Nature Communications 16: 3284. 10.1038/s41467-025-58656-y PMC11973199 40189591

[nph71466-bib-0108] Marks RA , Farrant JM , Nicholas McLetchie D , VanBuren R . 2021a. Unexplored dimensions of variability in vegetative desiccation tolerance. American Journal of Botany 108: 346–358. 33421106 10.1002/ajb2.1588

[nph71466-bib-0109] Marks RA , Hotaling S , Frandsen PB , VanBuren R . 2021b. Representation and participation across 20 years of plant genome sequencing. Nature Plants 7: 1571–1578. 34845350 10.1038/s41477-021-01031-8 PMC8677620

[nph71466-bib-0110] Marks RA , Lovell JT , Carey SB , Van Der Pas L , Chimukuche NM , Brůna T , Plott C , Webber J , Lipzen A , Yan J *et al*. 2025b. The architecture of resilience: a genome assembly of *Myrothamnus flabellifolia*sheds Light on desiccation tolerance and sex determination. New Phytologist 249: 1063–1084. 41178124 10.1111/nph.70700 PMC12712429

[nph71466-bib-0111] Marks RA , Van Der Pas L , Schuster J , Gilman IS , VanBuren R . 2024b. Convergent evolution of desiccation tolerance in grasses. Nature Plants 10: 1112–1125. 38906996 10.1038/s41477-024-01729-5

[nph71466-bib-0112] McGinnis LK , Zhu L , Lawitts JA , Bhowmick S , Toner M , Biggers JD . 2005. Mouse sperm desiccated and stored in trehalose medium without freezing. Biology of Reproduction 73: 627–633. 15930320 10.1095/biolreprod.105.042291

[nph71466-bib-0113] Mihailova G , Tchorbadjieva M , Rakleova G , Georgieva K . 2023. Differential accumulation of sHSPs isoforms during desiccation of the resurrection plant *Haberlea rhodopensis* Friv. under optimal and high temperature. Life 13: 238. 36676187 10.3390/life13010238 PMC9863180

[nph71466-bib-0114] Montes RAC , Haber A , Pardo J , Powell RF , Divisetty UK , Silva AT , Hernández‐Hernández T , Silveira V , Tang H , Lyons E *et al*. 2022. A comparative genomics examination of desiccation tolerance and sensitivity in two sister grass species. Proceedings of the National Academy of Sciences, USA 119: e2118886119. 10.1073/pnas.2118886119 PMC8812550 35082155

[nph71466-bib-0115] Moore JP , Hearshaw M , Ravenscroft N , Lindsey GG , Farrant JM , Brandt WF . 2007. Desiccation‐induced ultrastructural and biochemical changes in the leaves of the resurrection plant *Myrothamnus flabellifolia* . Australian Journal of Botany 55: 482.

[nph71466-bib-0116] Moore JP , Le NT , Brandt WF , Driouich A , Farrant JM . 2009. Towards a systems‐based understanding of plant desiccation tolerance. Trends in Plant Science 14: 110–117. 19179102 10.1016/j.tplants.2008.11.007

[nph71466-bib-0117] Morris JL , Puttick MN , Clark JW , Edwards D , Kenrick P , Pressel S , Wellman CH , Yang Z , Schneider H , Donoghue PCJ . 2018. The timescale of early land plant evolution. Proceedings of the National Academy of Sciences, USA 115: E2274–E2283. 10.1073/pnas.1719588115 PMC5877938 29463716

[nph71466-bib-0118] Müller J , Sprenger N , Bortlik K , Boller T , Wiemken A . 1997. Desiccation increases sucrose levels in *Ramonda* and *Haberlea*, two genera of resurrection plants in the Gesneriaceae. Physiologia Plantarum 100: 153–158.

[nph71466-bib-0119] Nesmelov A , Cornette R , Gusev O , Kikawada T . 2018. The antioxidant system in the anhydrobiotic midge as an essential, adaptive mechanism for desiccation survival. Advances in Experimental Medicine and Biology 1081: 259–270. 30288714 10.1007/978-981-13-1244-1_14

[nph71466-bib-0120] Neumann S , Reuner A , Brümmer F , Schill RO . 2009. DNA damage in storage cells of anhydrobiotic tardigrades. Comparative Biochemistry and Physiology. Part A, Molecular & Integrative Physiology 153: 425–429. 10.1016/j.cbpa.2009.04.611 19361569

[nph71466-bib-0121] Nguyen K , Kc S , Gonzalez T , Tapia H , Boothby TC . 2022. Trehalose and tardigrade CAHS proteins work synergistically to promote desiccation tolerance. Communications Biology 5: 1046. 36182981 10.1038/s42003-022-04015-2 PMC9526748

[nph71466-bib-0122] Nguyen K‐H , Chollet‐Krugler M , Gouault N , Tomasi S . 2013. UV‐protectant metabolites from lichens and their symbiotic partners. Natural Product Reports 30: 1490–1508. 24170172 10.1039/c3np70064j

[nph71466-bib-0123] Nishimura N , Hitomi K , Arvai AS , Rambo RP , Hitomi C , Cutler SR , Schroeder JI , Getzoff ED . 2009. Structural mechanism of abscisic acid binding and signaling by dimeric PYR1. Science 326: 1373–1379. 19933100 10.1126/science.1181829 PMC2835493

[nph71466-bib-0124] Nishizawa A , Yabuta Y , Shigeoka S . 2008. Galactinol and raffinose constitute a novel function to protect plants from oxidative damage. Plant Physiology 147: 1251–1263. 18502973 10.1104/pp.108.122465 PMC2442551

[nph71466-bib-0125] Oliver MJ , Farrant JM , Hilhorst HWM , Mundree S , Williams B , Bewley JD . 2020. Desiccation tolerance: avoiding cellular damage during drying and rehydration. Annual Review of Plant Biology 71: 435–460. 10.1146/annurev-arplant-071219-105542 32040342

[nph71466-bib-0126] Oliver MJ , Tuba Z , Mishler BD . 2000. The evolution of vegetative desiccation tolerance in land plants. Plant Ecology 151: 85–100.

[nph71466-bib-0127] Ooms J , Leon‐Kloosterziel KM , Bartels D , Koornneef M , Karssen CM . 1993. Acquisition of desiccation tolerance and longevity in seeds of *Arabidopsis thaliana* (a comparative study using abscisic acid‐insensitive abi3 mutants). Plant Physiology 102: 1185–1191. 12231895 10.1104/pp.102.4.1185 PMC158904

[nph71466-bib-0128] Ostria‐Gallardo E , Larama G , Berríos G , Fallard A , Gutiérrez‐Moraga A , Ensminger I , Bravo LA . 2020. A comparative gene co‐expression analysis using self‐organizing maps on two congener filmy ferns identifies specific desiccation tolerance mechanisms associated to their microhabitat preference. BMC Plant Biology 20: 56. 32019526 10.1186/s12870-019-2182-3 PMC7001327

[nph71466-bib-0129] Pammenter NW , Berjak P . 1999. A review of recalcitrant seed physiology in relation to desiccation‐tolerance mechanisms. Seed Science Research 9: 13–37.

[nph71466-bib-0130] Pardo J . 2022. Evolution of drought and desiccation tolerance in grasses. East Lansing, MI, USA: Michigan State University.

[nph71466-bib-0131] Pardo J , Man Wai C , Chay H , Madden CF , Hilhorst HWM , Farrant JM , VanBuren R . 2020. Intertwined signatures of desiccation and drought tolerance in grasses. Proceedings of the National Academy of Sciences, USA 117: 10079–10088. 10.1073/pnas.2001928117 PMC7211927 32327609

[nph71466-bib-0132] Parreira JR , Balestrazzi A , Fevereiro P , Araújo S d S . 2018. Maintaining genome integrity during seed development in *Phaseolus vulgaris* L.: evidence from a transcriptomic profiling study. Genes 9: 463. 30241355 10.3390/genes9100463 PMC6209899

[nph71466-bib-0133] Parsell DA , Lindquist S . 1993. The function of heat‐shock proteins in stress tolerance: degradation and reactivation of damaged proteins. Annual Review of Genetics 27: 437–496. 10.1146/annurev.ge.27.120193.002253 8122909

[nph71466-bib-0134] Peers G , Truong TB , Ostendorf E , Busch A , Elrad D , Grossman AR , Hippler M , Niyogi KK . 2009. An ancient light‐harvesting protein is critical for the regulation of algal photosynthesis. Nature 462: 518–521. 19940928 10.1038/nature08587

[nph71466-bib-0135] Peredo EL , Cardon ZG . 2020. Shared up‐regulation and contrasting down‐regulation of gene expression distinguish desiccation‐tolerant from intolerant green algae. Proceedings of the National Academy of Sciences, USA 117: 17438–17445. 10.1073/pnas.1906904117 PMC7382218 32636259

[nph71466-bib-0136] Phillips JR , Fischer E , Baron M , van den Dries N , Facchinelli F , Kutzer M , Rahmanzadeh R , Remus D , Bartels D . 2008. *Lindernia brevidens*: a novel desiccation‐tolerant vascular plant, endemic to ancient tropical rainforests. The Plant Journal 54: 938–948. 18346195 10.1111/j.1365-313X.2008.03478.x

[nph71466-bib-0137] Phillips JR , Hilbricht T , Salamini F , Bartels D . 2002. A novel abscisic acid‐ and dehydration‐responsive gene family from the resurrection plant *Craterostigma plantagineum* encodes a plastid‐targeted protein with DNA‐binding activity. Planta 215: 258–266. 12029475 10.1007/s00425-002-0755-z

[nph71466-bib-0138] Pogson BJ , Albrecht V . 2011. Genetic dissection of chloroplast biogenesis and development: an overview. Plant Physiology 155: 1545–1551. 21330494 10.1104/pp.110.170365 PMC3091115

[nph71466-bib-0139] Porembski S . 2007. Tropical inselbergs: habitat types, adaptive strategies and diversity patterns. Brazilian Journal of Botany 30: 579–586.

[nph71466-bib-0140] Porembski S . 2011. Evolution, diversity, and habitats of poikilohydrous vascular plants. In: Bartels D , Lüttge U , Beck E , eds. Plant desiccation tolerance. Berlin, Heidelberg, Germany: Springer, 139–156.

[nph71466-bib-0141] Porembski S , Barthlott W . 2000. Granitic and gneissic outcrops (inselbergs) as centers of diversity for desiccation‐tolerant vascular plants. Plant Ecology 151: 19–28.

[nph71466-bib-0142] Porembski S , Rexroth J , Weising K , Bondi L , Mello‐Silva R , Centeno DC , Datar MN , Watve A , Thiombano A , Tindano E *et al*. 2021. An overview on desiccation‐tolerant mat‐forming monocotyledons on tropical inselbergs. Flora 285: 151953.

[nph71466-bib-0143] Potts M . 1994. Desiccation tolerance of prokaryotes. Microbiological Reviews 58: 755–805. 7854254 10.1128/mr.58.4.755-805.1994 PMC372989

[nph71466-bib-0144] Potts M . 1999. Mechanisms of desiccation tolerance in cyanobacteria. European Journal of Phycology 34: 319–328.

[nph71466-bib-0145] Potts M , Slaughter SM , Hunneke F‐U , Garst JF , Helm RF . 2005. Desiccation tolerance of prokaryotes: application of principles to human cells. Integrative and Comparative Biology 45: 800–809. 21676831 10.1093/icb/45.5.800

[nph71466-bib-0146] Proctor MCF , Oliver MJ , Wood AJ , Alpert P , Stark LR . 2007. Desiccation‐tolerance in bryophytes: a review. The Bryologist 110: 595–621.

[nph71466-bib-0147] Proctor MCF , Tuba Z . 2002. Poikilohydry and homoihydry: antithesis or spectrum of possibilities? New Phytologist 156: 327–349. 33873572 10.1046/j.1469-8137.2002.00526.x

[nph71466-bib-0148] Rafsanjani A , Brulé V , Western TL , Pasini D . 2015. Hydro‐responsive curling of the resurrection plant *Selaginella lepidophylla* . Scientific Reports 5: 8064. 25623361 10.1038/srep08064 PMC4306918

[nph71466-bib-0149] Rastogi RP , Sonani RR , Madamwar D . 2015. Cyanobacterial sunscreen scytonemin: role in photoprotection and biomedical research. Applied Biochemistry and Biotechnology 176: 1551–1563. 26013282 10.1007/s12010-015-1676-1

[nph71466-bib-0150] Ratnakumar S , Hesketh A , Gkargkas K , Wilson M , Rash BM , Hayes A , Tunnacliffe A , Oliver SG . 2011. Phenomic and transcriptomic analyses reveal that autophagy plays a major role in desiccation tolerance in *Saccharomyces cerevisiae* . Molecular BioSystems 7: 139–149. 20963216 10.1039/c0mb00114g

[nph71466-bib-0151] Rebecchi L . 2013. Dry up and survive: the role of antioxidant defences in anhydrobiotic organisms. Journal of Limnology 72: 62–72.

[nph71466-bib-0152] Reina‐Bueno M , Argandoña M , Nieto JJ , Hidalgo‐García A , Iglesias‐Guerra F , Delgado MJ , Vargas C . 2012. Role of trehalose in heat and desiccation tolerance in the soil bacterium *Rhizobium etli* . BMC Microbiology 12: 207. 22985230 10.1186/1471-2180-12-207 PMC3518184

[nph71466-bib-0153] Richter K , Haslbeck M , Buchner J . 2010. The heat shock response: life on the verge of death. Molecular Cell 40: 253–266. 20965420 10.1016/j.molcel.2010.10.006

[nph71466-bib-0154] Rippin M , Becker B , Holzinger A . 2017. Enhanced desiccation tolerance in mature cultures of the streptophytic green alga *Zygnema circumcarinatum* revealed by transcriptomics. Plant & Cell Physiology 58: 2067–2084. 29036673 10.1093/pcp/pcx136 PMC5722205

[nph71466-bib-0155] Rizzo AM , Negroni M , Altiero T , Montorfano G , Corsetto P , Berselli P , Berra B , Guidetti R , Rebecchi L . 2010. Antioxidant defences in hydrated and desiccated states of the tardigrade *Paramacrobiotus richtersi* . Comparative Biochemistry and Physiology. Part B, Biochemistry & Molecular Biology 156: 115–121. 10.1016/j.cbpb.2010.02.009 20206711

[nph71466-bib-0156] Rodriguez MCS , Edsgärd D , Hussain SS , Alquezar D , Rasmussen M , Gilbert T , Nielsen BH , Bartels D , Mundy J . 2010. Transcriptomes of the desiccation‐tolerant resurrection plant *Craterostigma plantagineum* . The Plant Journal 63: 212–228. 20444235 10.1111/j.1365-313X.2010.04243.x

[nph71466-bib-0157] Roodt R , Spies JJ . 2003. Chromosome studies in the grass subfamily Chloridoideae. II. An analysis of polyploidy. Taxon 52: 736–746.

[nph71466-bib-0158] Rottet S , Besagni C , Kessler F . 2015. The role of plastoglobules in thylakoid lipid remodeling during plant development. Biochimica et Biophysica Acta 1847: 889–899. 25667966 10.1016/j.bbabio.2015.02.002

[nph71466-bib-0159] Rubinstein CV , Gerrienne P , de la Puente GS , Astini RA , Steemans P . 2010. Early middle Ordovician evidence for land plants in Argentina (eastern Gondwana): rapid report. New Phytologist 188: 365–369. 20731783 10.1111/j.1469-8137.2010.03433.x

[nph71466-bib-0160] Schwab KB , Schreiber U , Heber U . 1989. Response of photosynthesis and respiration of resurrection plants to desiccation and rehydration. Planta 177: 217–227. 24212344 10.1007/BF00392810

[nph71466-bib-0161] Sherwin HW , Farrant JM . 1998. Protection mechanisms against excess light in the resurrection plants Craterostigma wilmsii and *Xerophyta viscosa* . Plant Growth Regulation 24: 203–210.

[nph71466-bib-0162] Silva AT , Gao B , Fisher KM , Mishler BD , Ekwealor JTB , Stark LR , Li X , Zhang D , Bowker MA , Brinda JC *et al*. 2021. To dry perchance to live: insights from the genome of the desiccation‐tolerant biocrust moss *Syntrichia caninervis* . The Plant Journal 105: 1339–1356. 33277766 10.1111/tpj.15116

[nph71466-bib-0163] Singh SP , Häder D‐P , Sinha RP . 2010. Cyanobacteria and ultraviolet radiation (UVR) stress: mitigation strategies. Ageing Research Reviews 9: 79–90. 19524071 10.1016/j.arr.2009.05.004

[nph71466-bib-0164] Smrithy V , Kulkarni A , Shigwan BK , Porembski S , Datar MN . 2023. Desiccation‐tolerant vascular plants from Western Ghats, India: review, updated checklist, future prospects and new insights. Nordic Journal of Botany 5: e03939.

[nph71466-bib-0165] Sochava IV , Smirnova OI . 1993. Heat capacity of hydrated and dehydrated globular proteins. Denaturation increment of heat capacity. Food Hydrocolloids 6: 513–524. 8487767

[nph71466-bib-0166] St Aubin B , Wai CM , Kenchanmane Raju SK , Niederhuth CE , VanBuren R . 2022. Regulatory dynamics distinguishing desiccation tolerance strategies within resurrection grasses. Plant Direct 6: e457. 36523607 10.1002/pld3.457 PMC9748243

[nph71466-bib-0167] Tapia H , Young L , Fox D , Bertozzi CR , Koshland D . 2015. Increasing intracellular trehalose is sufficient to confer desiccation tolerance to Saccharomyces cerevisiae. Proceedings of the National Academy of Sciences, USA 112: 6122–6127. 10.1073/pnas.1506415112 PMC4434740 25918381

[nph71466-bib-0168] Tebele SM , Marks RA , Farrant JM . 2021. Two decades of desiccation biology: a systematic review of the best studied angiosperm resurrection plants. Plants 10: 2784. 34961255 10.3390/plants10122784 PMC8706221

[nph71466-bib-0169] Tibiletti T , Auroy P , Peltier G , Caffarri S . 2016. *Chlamydomonas reinhardtii* PsbS protein is functional and accumulates rapidly and transiently under high light. Plant Physiology 171: 2717–2730. 27329221 10.1104/pp.16.00572 PMC4972282

[nph71466-bib-0170] Tóth S , Kiss C , Scott P , Kovács G , Sorvari S , Toldi O . 2006. Agrobacterium‐mediated genetic transformation of the desiccation tolerant resurrection plant *Ramonda myconi* (L.) Rchb. Plant Cell Reports 25: 442–449. 16362301 10.1007/s00299-005-0083-4

[nph71466-bib-0171] Tuba Z , Lichtenthaler HK . 2011. Ecophysiology of homoiochlorophyllous and poikilochlorophyllous desiccation‐tolerant plants and vegetations. In: Ecological studies: analysis and synthesis. Berlin, Heidelberg, New York. Plant desiccation tolerance. Berlin, Heidelberg, Germany: Springer Berlin Heidelberg, 157–183.

[nph71466-bib-0172] Tuba Z , Protor CF , Csintalan Z . 1998. Ecophysiological responses of homoiochlorophyllous and poikilochlorophyllous desiccation tolerant plants: a comparison and an ecological perspective. Plant Growth Regulation 24: 211–217.

[nph71466-bib-0173] Tunnacliffe A , Wise MJ . 2007. The continuing conundrum of the LEA proteins. The Science of Nature 94: 791–812. 10.1007/s00114-007-0254-y 17479232

[nph71466-bib-0174] Tyson T , O'Mahony Zamora G , Wong S , Skelton M , Daly B , Jones JT , Mulvihill ED , Elsworth B , Phillips M , Blaxter M *et al*. 2012. A molecular analysis of desiccation tolerance mechanisms in the anhydrobiotic nematode *Panagrolaimus superbus* using expressed sequenced tags. BMC Research Notes 5: 68. 22281184 10.1186/1756-0500-5-68 PMC3296651

[nph71466-bib-0175] Vaidya AS , Helander JDM , Peterson FC , Elzinga D , Dejonghe W , Kaundal A , Park S‐Y , Xing Z , Mega R , Takeuchi J *et al*. 2019. Dynamic control of plant water use using designed ABA receptor agonists. Science 366: eaaw8848. 31649167 10.1126/science.aaw8848

[nph71466-bib-0176] VanBuren R , Bryant D , Edger PP , Tang H , Burgess D , Challabathula D , Spittle K , Hall R , Gu J , Lyons E *et al*. 2015. Single‐molecule sequencing of the desiccation‐tolerant grass *Oropetium thomaeum* . Nature 527: 508–511. 26560029 10.1038/nature15714

[nph71466-bib-0177] VanBuren R , Man Wai C , Pardo J , Giarola V , Ambrosini S , Song X , Bartels D . 2018a. Desiccation tolerance evolved through gene duplication and network rewiring in Lindernia. Plant Cell 30: 2943–2958. 30361236 10.1105/tpc.18.00517 PMC6354263

[nph71466-bib-0178] VanBuren R , Pardo J , Man Wai C , Evans S , Bartels D . 2019. Massive tandem proliferation of ELIPs supports convergent evolution of desiccation tolerance across land plants. Plant Physiology 179: 1040–1049. 30602492 10.1104/pp.18.01420 PMC6393792

[nph71466-bib-0179] VanBuren R , Wai CM , Giarola V , Župunski M , Pardo J , Kalinowski M , Grossmann G , Bartels D . 2023. Core cellular and tissue‐specific mechanisms enable desiccation tolerance in Craterostigma. The Plant Journal 114: 231–245. 36843450 10.1111/tpj.16165

[nph71466-bib-0180] VanBuren R , Wai CM , Keilwagen J , Pardo J . 2018b. A chromosome‐scale assembly of the model desiccation tolerant grass *Oropetium thomaeum* . Plant Direct 2: e00096. 31245697 10.1002/pld3.96 PMC6508818

[nph71466-bib-0181] VanBuren R , Wai CM , Ou S , Pardo J , Bryant D , Jiang N , Mockler TC , Edger P , Michael TP . 2018c. Extreme haplotype variation in the desiccation‐tolerant clubmoss *Selaginella lepidophylla* . Nature Communications 9: 13. 10.1038/s41467-017-02546-5 PMC5750206 29296019

[nph71466-bib-0182] VanBuren R , Wai CM , Zhang Q , Song X , Edger PP , Bryant D , Michael TP , Mockler TC , Bartels D . 2017. Seed desiccation mechanisms co‐opted for vegetative desiccation in the resurrection grass *Oropetium thomaeum* . Plant, Cell & Environment 40: 2292–2306. 10.1111/pce.13027 28730594

[nph71466-bib-0183] Vander Willigen C , Pammenter NW , Mundree S , Farrant J . 2001. Some physiological comparisons between the resurrection grass, *Eragrostis nindensis*, and the related desiccation‐sensitive species, *E. curvula* . Plant Growth Regulation 35: 121–129.

[nph71466-bib-0184] Vanschoenwinkel B , de Paula LFA , Snoeks JM , Van der Stocken T , Buschke FT , Porembski S , Silveira FAO . 2025. The ecological and evolutionary dynamics of inselbergs. Biological Reviews of the Cambridge Philosophical Society 100: 481–507. 39352171 10.1111/brv.13150

[nph71466-bib-0185] de Vries J , Archibald JM . 2018. Plant evolution: landmarks on the path to terrestrial life. New Phytologist 217: 1428–1434. 29318635 10.1111/nph.14975

[nph71466-bib-0186] Walters C , Hill LM , Wheeler LJ . 2005. Dying while dry: kinetics and mechanisms of deterioration in desiccated organisms. Integrative and Comparative Biology 45: 751–758. 21676826 10.1093/icb/45.5.751

[nph71466-bib-0187] Wang Y , Jiang J , Zhao X , Liu G , Yang C , Zhan L . 2006. A novel LEA gene from *Tamarix androssowii* confers drought tolerance in transgenic tobacco. Plant Science 171: 655–662.

[nph71466-bib-0188] Wang Y , Zhu L , Yang Y , Li X , Zhang X , Fang X . 2026. Cellular water‐potential sensing through biomolecular condensation. Nature. doi: 10.1038/s41586-026-10591-8. PMC13421324 42203875

[nph71466-bib-0189] Watanabe M . 2006. Anhydrobiosis in invertebrates. Applied Entomology and Zoology 41: 15–31.

[nph71466-bib-0190] Waterworth WM , Bray CM , West CE . 2019. Seeds and the art of genome maintenance. Frontiers in Plant Science 10: 706. 31214224 10.3389/fpls.2019.00706 PMC6554324

[nph71466-bib-0191] Wehmeyer N , Vierling E . 2000. The expression of small heat shock proteins in seeds responds to discrete developmental signals and suggests a general protective role in desiccation tolerance. Plant Physiology 122: 1099–1108. 10759505 10.1104/pp.122.4.1099 PMC58944

[nph71466-bib-0192] Whittaker A , Bochicchio A , Vazzana C , Lindsey G , Farrant J . 2001. Changes in leaf hexokinase activity and metabolite levels in response to drying in the desiccation‐tolerant species *Sporobolus stapfianus* and *Xerophyta viscosa* . Journal of Experimental Botany 52: 961–969. 11432913 10.1093/jexbot/52.358.961

[nph71466-bib-0193] Whittaker A , Martinelli T , Farrant JM , Bochicchio A , Vazzana C . 2007. Sucrose phosphate synthase activity and the co‐ordination of carbon partitioning during sucrose and amino acid accumulation in desiccation‐tolerant leaf material of the C4 resurrection plant *Sporobolus stapfianus* during dehydration. Journal of Experimental Botany 58: 3775–3787. 18057046 10.1093/jxb/erm228

[nph71466-bib-0194] Williams B , Njaci I , Moghaddam L , Long H , Dickman MB , Zhang X , Mundree S . 2015. Trehalose accumulation triggers autophagy during plant desiccation. PLoS Genetics 11: e1005705. 26633550 10.1371/journal.pgen.1005705 PMC4669190

[nph71466-bib-0195] Wise MJ , Tunnacliffe A . 2004. POPP the question: what do LEA proteins do? Trends in Plant Science 9: 13–17. 14729214 10.1016/j.tplants.2003.10.012

[nph71466-bib-0196] Wolkers WF , McCready S , Brandt WF , Lindsey GG , Hoekstra FA . 2001. Isolation and characterization of a D‐7 LEA protein from pollen that stabilizes glasses *in vitro* . Biochimica et Biophysica Acta 1544: 196–206. 11341929 10.1016/s0167-4838(00)00220-x

[nph71466-bib-0197] Wolkers WF , Oldenhof H , Alberda M , Hoekstra FA . 1998. A Fourier transform infrared microspectroscopy study of sugar glasses: application to anhydrobiotic higher plant cells. Biochimica et Biophysica Acta 1379: 83–96. 9468336 10.1016/s0304-4165(97)00085-8

[nph71466-bib-0198] Womersley C . 1981. Biochemical and physiological aspects of anhydrobiosis. Comparative Biochemistry and Physiology Part B: Comparative Biochemistry 70: 669–678.

[nph71466-bib-0199] Wood AJ . 2007. The nature and distribution of vegetative desiccation‐tolerance in hornworts, liverworts and mosses. The Bryologist 110: 163–177.

[nph71466-bib-0200] Wright DJ , Smith SC , Joardar V , Scherer S , Jervis J , Warren A , Helm RF , Potts M . 2005. UV irradiation and desiccation modulate the three‐dimensional extracellular matrix of *Nostoc commune* (Cyanobacteria). The Journal of Biological Chemistry 280: 40271–40281. 16192267 10.1074/jbc.M505961200

[nph71466-bib-0201] Wright PE , Dyson HJ . 2015. Intrinsically disordered proteins in cellular signalling and regulation. Nature Reviews. Molecular Cell Biology 16: 18–29. 25531225 10.1038/nrm3920 PMC4405151

[nph71466-bib-0202] Xiao B , Huang Y , Tang N , Xiong L . 2007. Over‐expression of a LEA gene in rice improves drought resistance under the field conditions. Theoretical and Applied Genetics 115: 35–46. 17426956 10.1007/s00122-007-0538-9

[nph71466-bib-0203] Xiao L , Yang G , Zhang L , Yang X , Zhao S , Ji Z , Zhou Q , Hu M , Wang Y , Chen M *et al*. 2015. The resurrection genome of *Boea hygrometrica*: a blueprint for survival of dehydration. Proceedings of the National Academy of Sciences, USA 112: 5833–5837. 10.1073/pnas.1505811112 PMC4426394 25902549

[nph71466-bib-0204] Xu H‐F , Dai G‐Z , Bai Y , Shang J‐L , Zheng B , Ye D‐M , Shi H , Kaplan A , Qiu B‐S . 2022. Coevolution of tandemly repeated hlips and RpaB‐like transcriptional factor confers desiccation tolerance to subaerial *Nostoc* species. Proceedings of the National Academy of Sciences, USA 119: e2211244119. 10.1073/pnas.2211244119 PMC9586280 36215485

[nph71466-bib-0205] Xu Z , Xin T , Bartels D , Li Y , Gu W , Yao H , Liu S , Yu H , Pu X , Zhou J *et al*. 2018. Genome analysis of the ancient tracheophyte *Selaginella tamariscina* reveals evolutionary features relevant to the acquisition of desiccation tolerance. Molecular Plant 11: 983–994. 29777775 10.1016/j.molp.2018.05.003

[nph71466-bib-0206] Yu J , Lai Y , Wu X , Wu G , Guo C . 2016. Overexpression of OsEm1 encoding a group I LEA protein confers enhanced drought tolerance in rice. Biochemical and Biophysical Research Communications 478: 703–709. 27524243 10.1016/j.bbrc.2016.08.010

[nph71466-bib-0207] Yu J , Li L , Wang S , Dong S , Chen Z , Patel N , Goffinet B , Chen H , Liu H , Liu Y . 2020. Draft genome of the aquatic moss *Fontinalis antipyretica* (Fontinalaceae, Bryophyta). GigaByte 2020: gigabyte8. 36824590 10.46471/gigabyte.8 PMC9631980

[nph71466-bib-0208] Zamecnik J , Faltus M , Bilavcik A . 2021. Vitrification solutions for plant cryopreservation: Modification and properties. Plants 10: 2623. 34961099 10.3390/plants10122623 PMC8707230

[nph71466-bib-0209] Zhang Q , Song X , Bartels D . 2016. Enzymes and metabolites in carbohydrate metabolism of desiccation tolerant plants. Proteomes 4: 40. 28248249 10.3390/proteomes4040040 PMC5260972

[nph71466-bib-0210] Zhang X , Ekwealor JTB , Mishler BD , Silva AT , Yu L , Jones AK , Nelson ADL , Oliver MJ . 2024. *Syntrichia ruralis*: emerging model moss genome reveals a conserved and previously unknown regulator of desiccation in flowering plants. New Phytologist 243: 981–996. 38415863 10.1111/nph.19620

[nph71466-bib-0211] Zhuo C , Cai J , Guo Z . 2013. Overexpression of early light‐induced protein (ELIP) gene from *Medicago sativa* ssp. *falcata* increases tolerance to abiotic stresses. Agronomy Journal 105: 1433–1440.

[nph71466-bib-0212] Zia A , Walker BJ , Oung HMO , Charuvi D , Jahns P , Cousins AB , Farrant JM , Reich Z , Kirchhoff H . 2016. Protection of the photosynthetic apparatus against dehydration stress in the resurrection plant *Craterostigma pumilum* . The Plant Journal 87: 664–680. 27258321 10.1111/tpj.13227

